# Longitudinal fibre-specific white matter damage predicts cognitive decline in multiple sclerosis

**DOI:** 10.1093/braincomms/fcae018

**Published:** 2024-01-27

**Authors:** Ismail Koubiyr, Eva A Krijnen, Anand J C Eijlers, Iris Dekker, Hanneke E Hulst, Bernard M J Uitdehaag, Frederik Barkhof, Jeroen J G Geurts, Menno M Schoonheim

**Affiliations:** MS Center Amsterdam, Anatomy and Neurosciences, Amsterdam Neuroscience, Amsterdam UMC location VUmc, Amsterdam 1081 HV, The Netherlands; MS Center Amsterdam, Anatomy and Neurosciences, Amsterdam Neuroscience, Amsterdam UMC location VUmc, Amsterdam 1081 HV, The Netherlands; Department of Neurology, Massachusetts General Hospital, Harvard Medical School, Boston, MA 02114, United States; MS Center Amsterdam, Anatomy and Neurosciences, Amsterdam Neuroscience, Amsterdam UMC location VUmc, Amsterdam 1081 HV, The Netherlands; MS Center Amsterdam, Rehabilitation, Amsterdam Neuroscience, Amsterdam UMC location VUmc, Amsterdam 1081 HV, The Netherlands; Health, Medical and Neuropsychology Unit, Institute of Psychology, Leiden University, Leiden 2333 AK, The Netherlands; MS Center Amsterdam, Neurology, Amsterdam Neuroscience, Amsterdam UMC location VUmc, Amsterdam 1081 HV, The Netherlands; MS Center Amsterdam, Radiology and Nuclear Medicine, Amsterdam Neuroscience, Amsterdam UMC location VUmc, Amsterdam 1081 HV, The Netherlands; Queen Square Institute of Neurology and Centre for Medical Image Computing, University College London, London WC1V 6LJ, UK; MS Center Amsterdam, Anatomy and Neurosciences, Amsterdam Neuroscience, Amsterdam UMC location VUmc, Amsterdam 1081 HV, The Netherlands; MS Center Amsterdam, Anatomy and Neurosciences, Amsterdam Neuroscience, Amsterdam UMC location VUmc, Amsterdam 1081 HV, The Netherlands

**Keywords:** multiple sclerosis, MRI, diffusion MRI, cognition, longitudinal

## Abstract

During the course of multiple sclerosis, many patients experience cognitive deficits which are not simply driven by lesion number or location. By considering the full complexity of white matter structure at macro- and microstructural levels, our understanding of cognitive impairment in multiple sclerosis may increase substantially. Accordingly, this study aimed to investigate specific patterns of white matter degeneration, the evolution over time, the manifestation across different stages of the disease and their role in cognitive impairment using a novel fixel-based approach. Neuropsychological test scores and MRI scans including 30-direction diffusion-weighted images were collected from 327 multiple sclerosis patients (mean age = 48.34 years, 221 female) and 95 healthy controls (mean age = 45.70 years, 55 female). Of those, 233 patients and 61 healthy controls had similar follow-up assessments 5 years after. Patients scoring 1.5 or 2 standard deviations below healthy controls on at least two out of seven cognitive domains (from the Brief Repeatable Battery of Neuropsychological Tests, BRB-N) were classified as mildly cognitively impaired or cognitively impaired, respectively, or otherwise cognitively preserved. Fixel-based analysis of diffusion data was used to calculate fibre-specific measures (fibre density, reflecting microstructural diffuse axonal damage; fibre cross-section, reflecting macrostructural tract atrophy) within atlas-based white matter tracts at each visit. At baseline, all fixel-based measures were significantly worse in multiple sclerosis compared with healthy controls (*P* < 0.05). For both fibre density and fibre cross-section, a similar pattern was observed, with secondary progressive multiple sclerosis patients having the most severe damage, followed by primary progressive and relapsing–remitting multiple sclerosis. Similarly, damage was least severe in cognitively preserved (*n* = 177), more severe in mildly cognitively impaired (*n* = 63) and worst in cognitively impaired (*n* = 87; *P* < 0.05). Microstructural damage was most pronounced in the cingulum, while macrostructural alterations were most pronounced in the corticospinal tract, cingulum and superior longitudinal fasciculus. Over time, white matter alterations worsened most severely in progressive multiple sclerosis (*P* < 0.05), with white matter atrophy progression mainly seen in the corticospinal tract and microstructural axonal damage worsening in cingulum and superior longitudinal fasciculus. Cognitive decline at follow-up could be predicted by baseline fixel-based measures (*R*^2^ = 0.45, *P* < 0.001). Fixel-based approaches are sensitive to white matter degeneration patterns in multiple sclerosis and can have strong predictive value for cognitive impairment. Longitudinal deterioration was most marked in progressive multiple sclerosis, indicating that degeneration in white matter remains important to characterize further in this phenotype.

## Teaser

Koubiyr *et al*. report white matter (WM) degeneration in multiple sclerosis using a ‘fixel-based’ approach, which considers the full complexity of WM and shows higher sensitivity. Longitudinal deterioration was most marked in progressive multiple sclerosis, indicating that degeneration in WM remains important to characterize further in this phenotype.

## Graphical Abstract

**Graphical Abstract:**
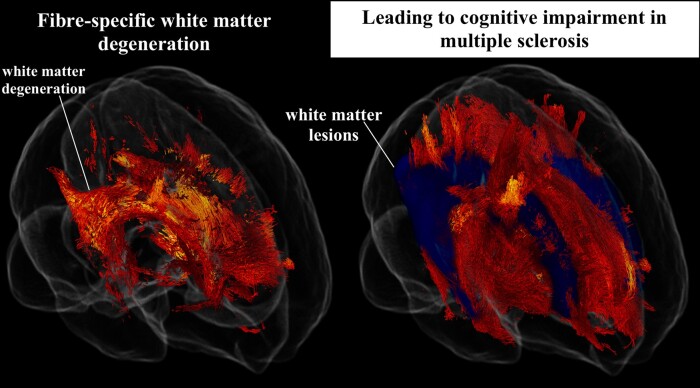

## Introduction

Multiple sclerosis (MS) has been primarily described as an inflammatory, demyelinating disease of the CNS. However, an increasing number of studies highlight the importance of neurodegeneration in MS pathology,^1^ such as neuro-axonal damage and loss.^2^

During the course of MS, many patients experience cognitive deficits^3^ profoundly impacting their quality of life.^4^ In recent years, many studies have shown an association between clinical disability, cognitive impairment (CI) and brain atrophy in MS.^5^ Some of those studies have demonstrated a greater contribution from grey matter (GM) atrophy compared with white matter (WM) atrophy in explaining cognitive deficits and disability worsening.^6-8^

Interestingly, WM atrophy appears clinically less relevant for patients.^9,10^ WM volume does not seem to parallel disease evolution or to explain clinical disability, with progressing and non-progressing patients having similar WM volumes^10^ and lack of association between WM atrophy and clinical worsening.^9,11^ So far, WM atrophy has been mainly assessed using conventional volumetric measures,^5^ which do not consider the full complexity of WM structure both at macro- and microstructural levels.

The importance of understanding white matter degeneration in MS is especially important given that MS is seen as a disconnection disease,^12^ where neuronal connections are damaged or even severed by lesions as well as diffuse axonal loss in tissues that appear normal on conventional MRI, all of whom are associated with cognitive dysfunction.^13^

Brain imaging methods based on MRI have investigated the neurodegenerative process in MS *in vivo* by measuring brain volume change, indicating strong clinical correlations.^5^ However, degeneration in the WM is more difficult to quantify *in vivo* using standard acquisitions, which could potentially explain the scarce associations between WM volumetric measures and clinic. Indeed, while neuronal death leads to a shrinkage of cortical GM, it is not necessarily the case that damage to the WM wiring also leads to a loss of volume in this compartment to the same degree but could also be partly replaced by scar tissue, for example, and could therefore go undetected by conventional volumetric assessments.

Diffusion-weighted imaging (DWI) is currently the only method available to investigate WM tract architecture *in vivo*. However, the significance of DWI studies is strongly linked to acquisition protocols, pre- and post-processing methods of data, quantitative analysis as well as careful interpretation of results.^14^ The majority of MS studies using DWI has been performed using the diffusion tensor model [diffusion tensor imaging (DTI)] and has investigated quantitative measures using voxel-based approaches derived from this model, such as fractional anisotropy and mean diffusivity, which aim to reflect the structural integrity of WM.^15^

Despite showing clear clinical correlations, DTI measures suffer from a limited ability to model complex multifibre voxels (e.g. with crossing-fibre populations), which may represent between 63% and 90% of WM voxels depending on the reconstruction algorithm.^16^ Thus, changes detected in WM voxels containing multifibre populations cannot be assigned to specific fibre pathways rendering them more difficult to interpret.^14^ In addition, DTI-based models cannot accurately describe reductions in fibre counts and as such cannot distinguish inflammatory and degenerative changes accurately. To circumvent these issues, fixel-based analysis (FBA) was proposed to investigate WM changes.^17^ This novel technique allows fibre tract-specific statistical analysis, where a ‘fixel’ refers to a specific fibre population within a voxel.^18^ This method is based on the analysis of DWI data using constrained spherical deconvolution (CSD),^19^ enabling us to characterize multiple fibre orientations within a given voxel and ultimately detect degeneration within specific WM tracts estimating:

- Fibre density (FD): A microstructural measure of the density of axons within a specific fibre population in a given voxel proportional to the intra-axonal volume. A decrease in FD could result from axonal loss.
- Fibre cross-section (FC): A morphological measure of the macroscopic change in the cross-sectional size of fibre bundles. A decrease in FC may be a consequence of WM atrophy induced by the narrowing of the extra-axonal space due to axonal loss or demyelination.
- Fibre density and cross-section (FDC): A combination of the two previous measures incorporating both micro- and macroscopic changes, sensitive to the capacity of the WM to transmit information.

Fixel-based approaches have been scarcely used in MS research^20-22^ and have not been related to physical and cognitive progression in large cohorts before. In this longitudinal study, we therefore aimed to investigate whether specific patterns of WM degeneration over time are specific for the different stages of the disease and how each pattern evolves over time in the context of clinical progression. Additionally, we investigated the predictive power of such novel patterns for disability progression and cognitive decline.

## Materials and methods

### Study protocol, approvals, registration and patient consent

The study was approved by the institutional ethics review board of the VU University Medical Center, and participants gave written informed consent prior to participation.

### Participants

In this longitudinal cohort study, we recruited 327 patients with clinically definite MS^23^ as part of the Amsterdam MS Cohort,^24^ as well as 95 healthy controls (HCs). At baseline, all subjects underwent an MRI exam and cognitive assessment. Disability was assessed in MS patients only using the Expanded Disability Status Scale (EDSS).^25^ An identical evaluation was performed at a second visit for 233/327 patients (71%) and 61/95 HC (64%), as the other subjects were lost to follow-up. Patients had a mean disease duration of 14.67 years at the first measurement. The average interval time between the two visits was 4.81 years for MS patients [standard deviation (SD) = 0.85] and 5.43 years for HC (SD = 1.07). Patient phenotypes included 230 relapsing–remitting (RRMS), 52 secondary progressive (SPMS) and 36 primary progressive MS (PPMS). Educational level was assessed according to the highest level attained. Disease-modifying treatments at baseline included β-interferons (*n* = 72), glatiramer acetate (*n* = 16), natalizumab (*n* = 22) or other immunosuppressive therapies (*n* = 6). Patients were relapse-free and without steroid treatment for at least 2 months prior to both baseline and follow-up visits. Part of the data included in this paper has been used in previous studies of our group investigating both the rate of GM atrophy^26^ and functional connectivity changes over time.^22,27^ The in-depth analysis of the WM has not been described before.

### Neuropsychological evaluation

Participants underwent a thorough neuropsychological assessment during both visits, using an expanded version of the Brief Repeatable Battery of Neuropsychological tests,^28^ as detailed previously.^29^ Verbal memory was measured through the selective reminding test, taking into account the average long-term storage from the initial trial, cumulative long-term recall and delayed recall scores. Verbal fluency was evaluated using the word list generation test, assessing the total number of accurate responses within a 60-s timeframe. Information processing speed was measured with the spoken version of the symbol digit modalities test, considering the overall number of correct responses within 90 s. Visuospatial memory was assessed through the spatial recall test, factoring in the total score from three consecutive recall trials and the delayed recall trial score. Attention was assessed using the Stroop colour-word test, incorporating the time taken to complete all three trials and the time taken to complete the third trial, adjusted for the time taken in the initial two trials. Working memory was evaluated through the memory comparison test, examining the time taken to complete each per cent sign, one-, two-, three- and four-letter trials. Lastly, executive functioning was assessed via the concept shifting test, involving alphabetical letter ordering, ascending number ordering and alternating letter and number ordering conditions, all adjusted for motor speed.

Subscores for each domain were converted into *Z*-scores based on the mean and SD of controls and averaged to create a unique *Z*-score per domain. The cognitive scores of all participants were corrected for effects of age, sex and education as described before,^8^ and *Z*-scores were calculated at each respective time point, thus considering change due to learning effects. Patients were then classified as cognitively impaired (CI; at least two out of seven domains with *Z* ≤ −2), mildly CI (MCI; at least two out of seven domains with *Z* ≤ −1.5 but not fulfilling CI criteria) and cognitively preserved (CP, not fulfilling CI or MCI criteria).^27^

### Magnetic resonance imaging

Participants were scanned at 3T (GE Signa-HDxt, Milwaukee, WI) using an eight-channel phased-array head coil. The acquisition protocol included a 3D T_1_-weighted fast spoiled gradient echo sequence for volumetric measurements and anatomical parcellation (repetition time, 7.8 ms; echo time, 3 ms; inversion time, 450 ms; flip angle, 12°, 0.9 × 0.9 mm^2^ in-plane resolution with 1 mm sagittal slices), a 3D fluid-attenuated inversion recovery (FLAIR) sequence for WM lesion segmentation (repetition time, 8000 ms; echo time, 125 ms; inversion time, 2350 ms, 1 × 1 mm^2^ in-plane resolution with 1.2 mm sagittal slices) and a diffusion-weighted echo-planar imaging sequence covering the whole brain with 5 volumes with *b* = 0 s/mm^2^ and 30 non-colinear directions with *b* = 1000 s/mm^2^ (repetition time, 13 000 ms; echo time, 91 ms; flip angle, 90°, 2 × 2 mm^2^ in-plane resolution with 53 contiguous axial slices of 2.4 mm).

### Conventional MRI processing

White matter lesions were automatically segmented on FLAIR images using k-nearest neighbour classification with tissue type priors.^30^ Using these maps, a lesion-filling algorithm was applied to the T_1_-weighted images to reduce the impact of lesions on brain tissue segmentation.^31^ Whole-brain, total WM and deep GM volumes were calculated using SIENAX^32^ and FIRST,^33^ both part of FSL5 (fsl.fmrib.ox.ac.uk). Cortical GM volume was determined by subtracting FIRST-based DGM segmentations from SIENAX-based total GM segmentations. All these volumes were normalized for head size using the V-scaling factor derived from SIENAX.

### Fixel-based analysis

FBA was implemented using MRtrix3 in accordance with the recommended pipeline.^17^ Preprocessing of diffusion-weighted data included denoising,^34^ eddy current and motion correction,^35^ bias field correction^36^ and up-sampling the spatial resolution in all three dimensions with cubic b-spline interpolation to a voxel size of 1.25  mm.^3,37^

We estimated the fibre orientation distribution (FOD) within each voxel using single-shell three-tissue CSD with group-averaged response functions for WM, GM and CSF^38^ followed by a global intensity normalization to correct for intensity inhomogeneities to enable quantitative group comparisons.^17^

A study-specific WM FOD template was created using the FOD-guided non-linear registration based on WM FODs from a subset of 20 HCs and 20 MS patients (MS patients were only included if their WM lesion volume or brain atrophy did not exceed 2 SDs above or below the mean, respectively, to ensure a representative sample as suggested previously^21^). Next, a longitudinal template was generated using previously described recommendations to reduce bias.^39^ For each participant, FOD maps of timepoints 1 and 2 were rigidly transformed to their midway space and then averaged to generate an unbiased intra-subject template. Then, all 40 intra-subject FOD templates were used to generate a longitudinal population template. For each participant, FOD maps of timepoints 1 and 2 were subsequently registered to this longitudinal population template and segmented to produce a set of discrete fixels. To enable connectivity-based fixel enhancement (CFE) for fixel statistics,^18^ a tractogram was generated using whole-brain probabilistic tractography on the population template. Twenty million streamlines were first generated and subsequently filtered to 2 million streamlines using the spherical deconvolution informed filtering of tractograms algorithm (SIFT1)^40^ to reduce reconstruction bias and improve biological plausibility.

Finally, fixel-specific measures of both FD and FC were calculated within each voxel (and log-transformed for FC, as recommended). We also derived the combined measure FDC by multiplying FD and FC.^17^

## Statistical analyses

### Demographic, clinical and conventional MRI data

Statistical analyses were performed using SPSS 26.0 (SPSS, Chicago, IL, USA). Normality of distribution was assessed by the Shapiro–Wilk test and visual inspection of the histograms. Normally distributed variables were compared between the HC, CP, MCI and CI groups at baseline with one-way ANOVAs for continuous measures (corrected for age, sex and education) or χ^2^ tests for categorical data. To use level of education as a covariate, this measure was transformed into a binary measure of high level of education, yes/no.

### Whole-brain fixel-based analysis

To identify regions with altered FD, FC and FDC in MS, we first compared these metrics using FBA at each white matter fixel across the brain using a general linear model (GLM) comparing MS patients versus HCs at baseline. Age and head size were entered as nuisance covariates.

Statistical inference was performed using the CFE method,^18^ a permutation-based, family-wise error-corrected *P*-value for every fixel in the template. Whole-brain FBA results reported were generated using 5000 permutations, and family-wise error-corrected statistical significance was set at *P*_FWE_ < 0.05. To better visualize the WM pathways implicated, significant fixels were displayed on the template-derived tractogram, in which streamlines were cropped to only include significant fixels. Streamlines were then colour-coded by the effect size expressed as a percentage relative to the control group.

### Tract-of-interest analysis

To investigate potential degeneration of specific fibre pathways along the disease course, we performed further tract-of-interest analyses. White matter tracts were categorized into 20 WM tracts using the Johns Hopkins University (JHU) DTI-based white matter atlas.^41^ For each tract containing fixels showing a significant change in the previous whole-brain analysis, the corresponding metric was averaged along all fixels belonging to this tract. The following comparisons were performed: (i) between the different disease phenotypes (RRMS, SPMS, PPMS) and HCs and (ii) between the different cognitive profiles (CP, MCI, CI) and HCs.

At baseline, groups were compared using a one-way ANOVA, corrected for age, sex and head size (education was also considered when comparing cognitive profiles). Over time, metrics were compared using linear mixed effect models with repeated measures to investigate the evolution of the different clinical phenotypes. All analyses were repeated after masking out WM lesions to evaluate their specific impact on results. All analyses were also repeated for patients with treatment as a confounding factor to control for its impact on our results. The treatment factor was considered in two different ways: whether a patient was under treatment or not (treatment = yes/no) or the type of treatment (treatment = β-interferons/glatiramer acetate/natalizumab/other/none). *P*-values < 0.05 were considered to indicate statistical significance after correcting for multiple models’ comparisons using Bonferroni correction. This statistical analysis was performed in R 3.6.3 and Python 3.7.3.

### Association with physical disability and cognition

First, we investigated how degeneration of the previously depicted WM tracts could explain disability (EDSS) and the averaged cognitive *Z*-score at baseline. Univariate correlations with clinical scores were first performed as a data reduction step in order to select tracts of interest, which were further entered into multivariate regression models with backward selection to identify the most relevant combination of tracts. This model included three hierarchical blocks to assess the added value of fixel measures beyond measures of atrophy and lesion load. In the first block, relevant demographical (age, sex and education) and clinical (symptom duration) covariates were regressed. In the second block, conventional MRI features (volumes of WM, DGM, CGM and lesion load) were added. In the final block, significant fixel metrics from the univariate analysis were entered.

Then, in order to evaluate the predictive power of these novel metrics for future clinical or cognitive decline, we implemented a prediction framework integrating feature selection using a nested 10-fold cross-validation strategy. First, data were randomly divided into a training set (80%) and test set (20%). The training set was submitted to feature selection and to determine the optimal final model, while the test set was used to estimate the accuracy of the model on an isolated set. This method allows to avoid leakage of test data as we validate our measures of prediction on a clean isolated data set during the fitting procedure on training data.^42^ In each outer loop of the 10-fold cross-validation, 1 fold was left out as testing subjects, while the remaining 9 folds were used as the training set, in which an inner 5-fold univariate linear regression was run to determine the optimal features and build the model. Features (predictors) that were significantly retrieved in all inner loops (100% consistency) were further entered into a stepwise linear regression model for the outer predicting loop. This was performed using the stepAIC function from the MASS package in R 3.6.3. Akaike information criterion (AIC) was used to retain the best model that explains the most variation in data while penalizing for models that use an excessive number of parameters. This function adds and removes predictor variables from a regression model until we find the set of features that produces the model with the lowest AIC value. Importantly, the order of variable entry does not influence the final model. The 10-fold outer cross-validation loop yielded a final model with statistically significant predictors. The root mean square error (RMSE) and explained variance (*R*^2^) of the model was reported. Finally, the optimal model was applied to the isolated test set to measure its accuracy in terms of RMSE.

## Results

### Demographic, clinical and cognitive data

Table 1 summarizes demographic, clinical and cognitive data for HC and patients with different cognitive profiles at baseline. Median EDSS increased from 3.0 at baseline to 3.5 (*P* < 0.001), and 22 RRMS patients converted to a secondary progressive form. At baseline, cognitive profiles consisted of 177 CP (54%), 63 MCI (19%) and 87 CI (27%) patients. At follow-up, distribution over the cognitive groups changed, with 106 CP (45%), 51 MCI (22%) and 73 CI (31%) patients. Over time, 32 CP patients deteriorated and converted to MCI (*n* = 22) or to CI (*n* = 10), while 13 patients improved and reverted from MCI to CP (*n* = 9) and from CI to CP (*n* = 4). Average cognitive performance in patients at baseline was −0.80 (SD = 0.95), which decreased significantly over time to −0.98 (SD = 0.96).

**Table 1:** Demographic, clinical and conventional MRI data at baseline for healthy controls and MS patients

	HC (*n* = 95)	MS (*n* = 327)	CP (*n* = 177)	MCI (*n* = 63)	CI (*n* = 87)	*P*-value
Sex ratio, F/M	55/40	221/106	129/48	40/23	52/35	**0.045**
Age, years	45.70 (10.35)	48.34 (10.95)	46.58 (10.34)	49.49 (12.18)	51.09 (10.66)	**0.001**
Level of education, high/low	56/39	149/178*	95/82	25/38	29/58	**0.001**
Symptom duration, years		14.67 (8.46)	13.69 (7.91)	14.13 (8.24)	17.05 (9.32)	**0.008**
Disease phenotype (RRMS/SPMS/PPMS)		239/52/36	144/21/12	45/6/12	50/25/12	**<0.001**
Treatment, IF-β/GA/NTZ/other/none		72/16/22/6/211	35/6/15/4/117	18/6/1/1/37	19/4/6/1/57	0.315
EDSS, median (range)		3 (0–8)	3 (0–8)	3 (0–8)	4 (2–8)	**<0.001**
Average cognition, *Z*-scores	0.00 (0.48)	−0.80 (0.95)***	−0.19 (0.47)	−1.00 (0.35)	−1.90 (0.92)	**<0.001**
Normalized WM lesion load, mL		14.41 (13.16)	10.68 (9.72)	14.29 (11.11)	22.09 (16.90)	**<0.001**
NBV, mL	1517.23 (66.67)	1451.12 (78.19)***	1476.19 (63.16)	1454.00 (70.73)	1398.02 (85.18)	**<0.001**
NWMV, mL	697.73 (30.82)	668.34 (35.49)***	675.21 (32.93)	670.40 (35.08)	652.87 (36.41)	**<0.001**
NCGMV, mL	780.39 (51.65)	748.07 (53.74)***	764.62 (44.64)	749.02 (50.58)	713.71 (57.15)	**<0.001**
NDGMV, mL	62.95 (3.73)	56.00 (6.84)***	58.51 (5.15)	56.06 (5.65)	50.86 (7.77)	**<0.001**

*P*-values in the last column correspond to the one-way analysis of variance between CP, MCI and CI. Bold values indicate significant findings (*P* < 0.05).

HC, healthy controls; MS, multiple sclerosis; CP, cognitively preserved; MCI, mildly cognitively impaired; CI, cognitively impaired; EDSS, Expanded Disability Status Scale; IF-β, interferon beta; GA, glatiramer acetate; NTZ, natalizumab; WM, white matter; NBV, normalized brain volume; NCGMV, normalized cortical grey matter volume; NDGMV, normalized deep grey matter volume; NWMV, normalized white matter volume.

*<0.05; ***<0.001.

### Whole-brain fixel-based analysis

#### Baseline

**Figure 1:**
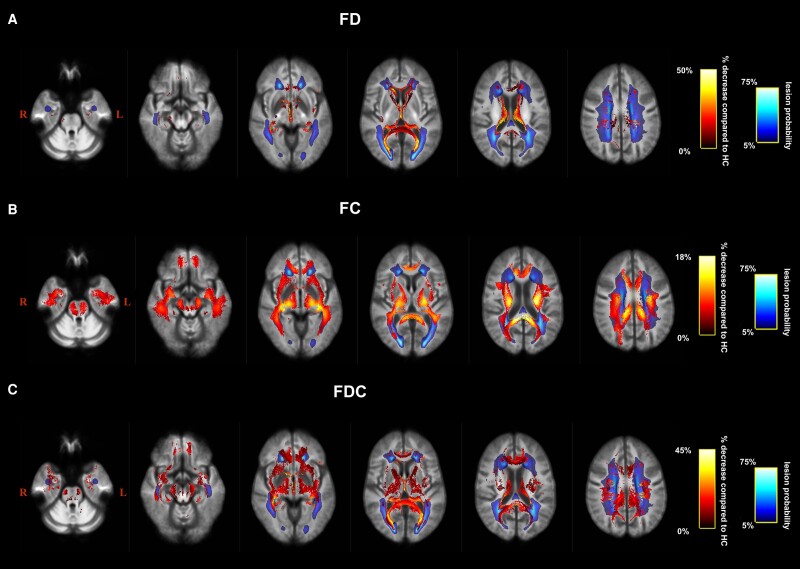
**Whole-brain fibre tract-specific reductions in multiple sclerosis patients at baseline**. Only streamline points that correspond to significant fixels were included (FWE-corrected *P*-value < 0.05). Streamlines were coloured by percentage effect decrease in the multiple sclerosis group compared with the HC group for (**A**) FD, (**B**) FC and **(C**) FDC. HC, healthy controls.

Whole-brain FBA results at baseline are summarized in Fig. 1A, B and C, displaying tracts containing fixels with (FWE-corrected *P*-value < 0.05) lower FD, fibre bundle cross-section and FDC in MS patients compared with HC. Microstructural FD decreases were observed across specific fibre pathways, especially across long association fibres such as the bilateral superior longitudinal fasciculus (SLF), the bilateral uncinate fasciculus, the bilateral inferior longitudinal fasciculus (ILF) and the bilateral cingulum. Other projection fibres [forceps major and corticospinal tract (CST)] were also altered but to a lesser extent (less altered fixels). Macrostructural decreases in fibre bundle cross-section were less pronounced (smaller effect sizes), though more spatially extensive. These partially overlapped with microstructural results and were mainly situated in long association fibres such as bilateral SLF and cingulum but also to a lesser extent the forceps minor. When we combined both micro- and macrostructural changes using FDC, the largest decreases occupied mainly the bilateral SLF, ILF and inferior fronto-occipital fasciculus (IFOF), with additional alterations in the bilateral anterior thalamic radiation and the forceps major. A lesion frequency map was overlaid over the altered fixels for each measure (Fig. 1A, B and C). Although some overlap was shown, the majority of streamline segments associated with altered fixels was spatially independent from lesions.

### Tract-of-interest analysis

#### Comparison between clinical phenotypes

##### Baseline

**Figure 2:**
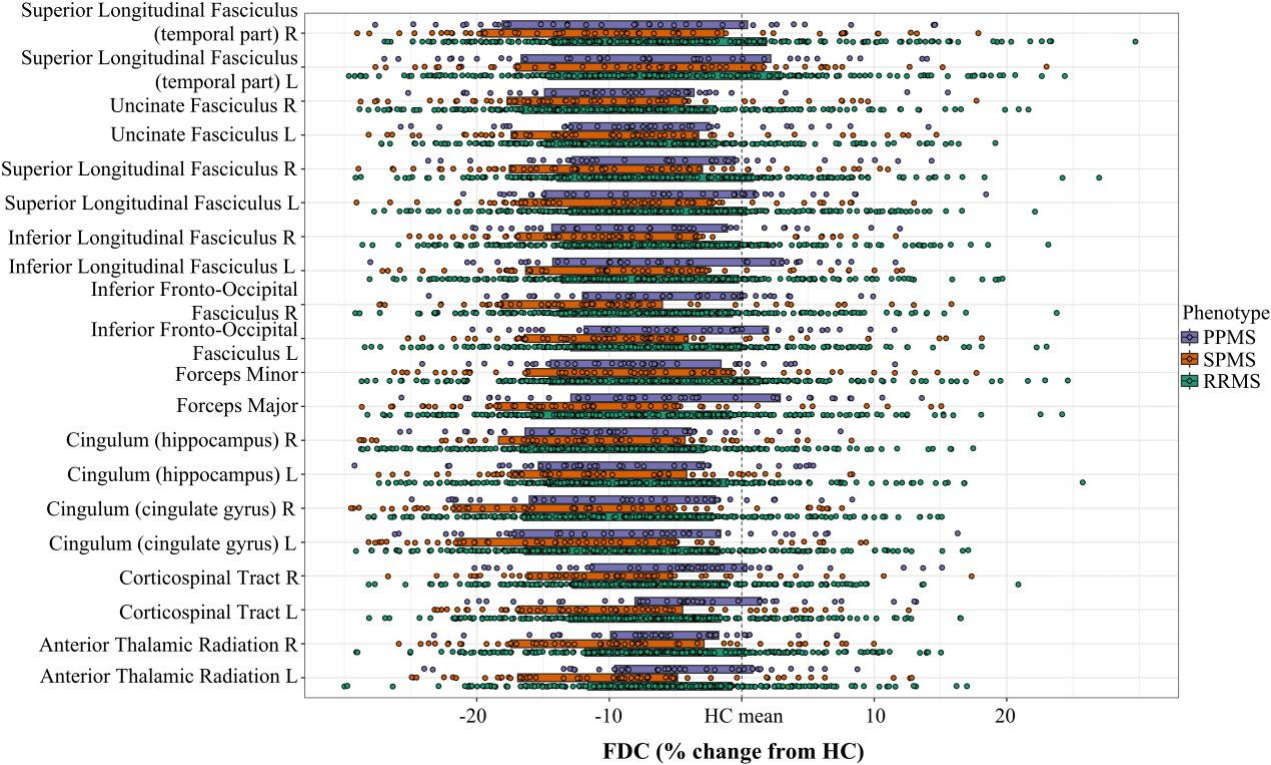
**Significant tracts in MS from baseline FDC tract-of-interest analysis comparing clinical phenotypes**. Mean FDC (diamonds) and 95% CIs (bars) within significant tracts (one-way ANOVAs, all *P*-values < 0.001, all *F* > 6.5) are displayed for PPMS, SPMS and RRMS groups, as a percentage difference from the HC mean. PPMS, primary progressive multiple sclerosis; SPMS, secondary progressive multiple sclerosis; RRMS, relapsing–remitting multiple sclerosis; HC, healthy controls; FDC, fibre density and cross-section; CIs, confidence intervals; L, left; R, right.

All tracts that showed significant differences between groups (clinical phenotypes) are reported in Fig. 2 and Supplementary Figs. 1 and 2 for FDC, FD and FC, respectively. First, we observed a pattern common to the three measures, where SPMS had the highest alterations among the groups, followed by PPMS which often showed similar damage compared with RRMS. Comparisons between pairs of groups in a post-hoc analysis are reported in full in Supplementary Tables 1, 2 and 3 and showed significant differences between HC and the different phenotypes, as well as between SPMS and the other phenotypes (all *P*-values < 0.05 and Bonferroni corrected). No differences were detected between RRMS and PPMS. Microstructural damage was most pronounced in the bilateral parahippocampal cingulum bundle, while macrostructural alterations were most pronounced in the bilateral CST, bilateral cingulum and SLF. As for the FDC, bilateral cingulum, bilateral anterior thalamic radiations and forceps major and minor showed the most extensive damage. When white matter lesions were masked out, results remained similar with a slight increase in effect sizes (Supplementary Figs. 3, 4 and 5). When repeating the analyses for patients, results were similar when including treatment information as a cofounding factor.

##### Changes over time

Mean values of FD decreased significantly over time, especially in progressive MS (Supplementary Fig. 6). Tracts that showed decline over time included bilateral cingulum (*P* = 0.029; RRMS, PPMS and SPMS), forceps minor (*P* < 0.001; RRMS, PPMS and SPMS), right ILF (*P* = 0.013; PPMS), left SLF (*P* = 0.005; PPMS and SPMS), right SLF (*P* < 0.001; PPMS) and right temporal part of SLF (*P* < 0.001; RRMS, PPMS and SPMS). For FC, only left CST (*P* = 0.003; SPMS) decreased significantly over time (Supplementary Fig. 7). As for FDC values (Supplementary Fig. 8), significant decrease was observed within left CST (*P* = 0.005; PPMS and SPMS), forceps major (*P* = 0.004; SPMS) and right ILF (*P* = 0.006; PPMS).

**Figure 3:**
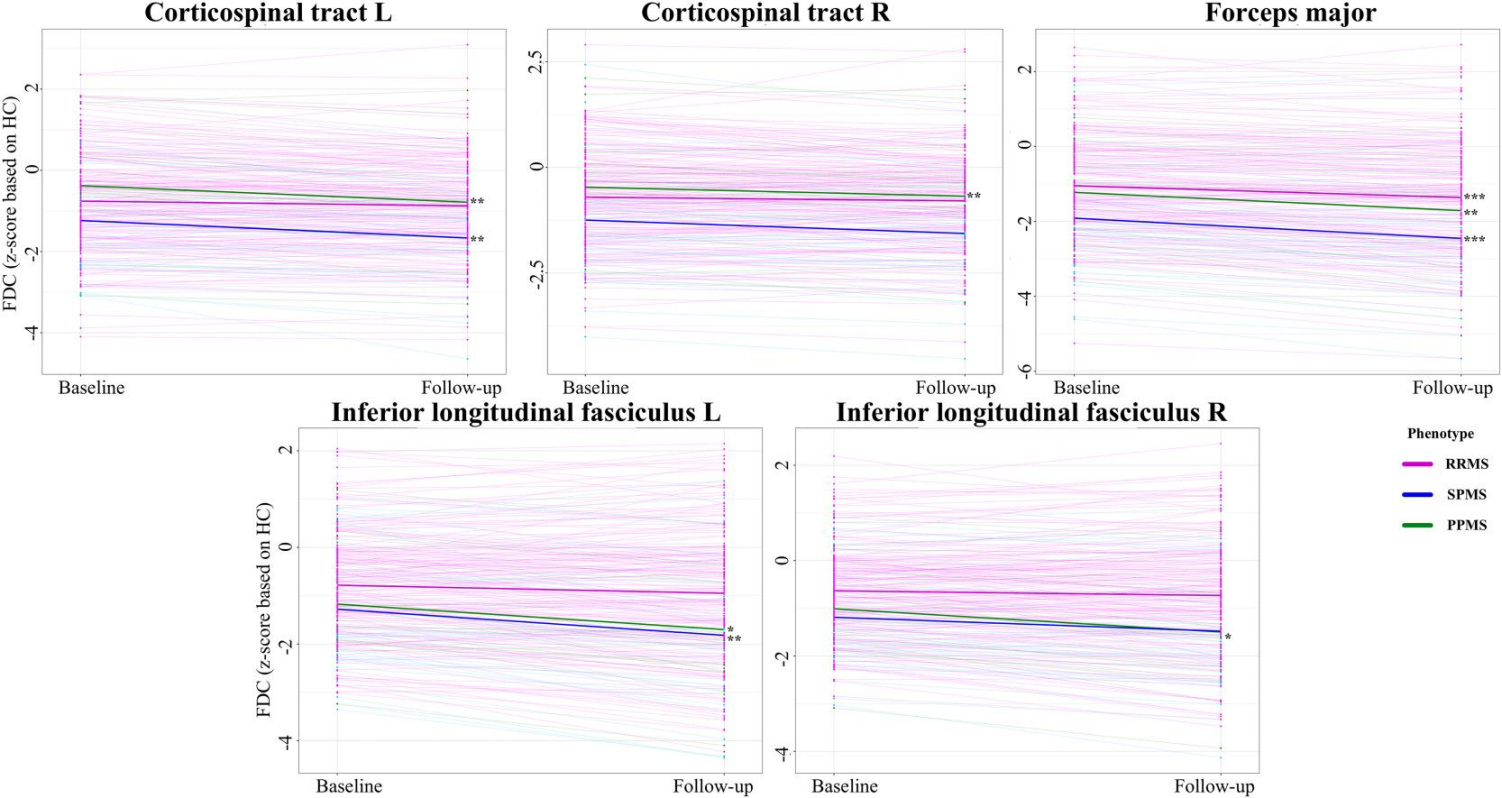
**Tracts with significant longitudinal changes of FDC by clinical phenotype**. Linear mixed effect models were used to investigate the longitudinal change of FDC over time (all *P*-values < 0.01, all *F* > 6.3). FDC, fibre density and cross-section; L, left; R, right. *<0.05; **<0.01; ***<0.001 indicate *P*-values for post-hoc analysis of longitudinal change per phenotype.

When white matter lesions were discarded, additional tracts showed significant change over time, again mostly in progressive MS. FD decreases (Supplementary Fig. 9) were now also observed in bilateral anterior thalamic radiations (left, *P* = 0.005; right, *P* = 0.001; PPMS and SPMS), left CST (*P* = 0.001; PPMS and SPMS), right parahippocampal cingulum (*P* = 0.004; PPMS), forceps major (*P* < 0.001; PPMS and SPMS), left IFOF (*P* < 0.001; PPMS and SPMS) and right IFOF (*P* < 0.001; RRMS, PPMS and SPMS). For FDC (Fig. 3), decreases over time were also seen in right CST (*P* = 0.042; PPMS), forceps major (*P* < 0.001; RRMS, PPMS and SPMS) and left ILF (*P* = 0.002; PPMS and SPMS). No additional tracts were found for FC (Supplementary Fig. 10). Results were similar when including treatment information as a cofounding factor.

#### Comparison between cognitive profiles

##### Baseline

**Figure 4:**
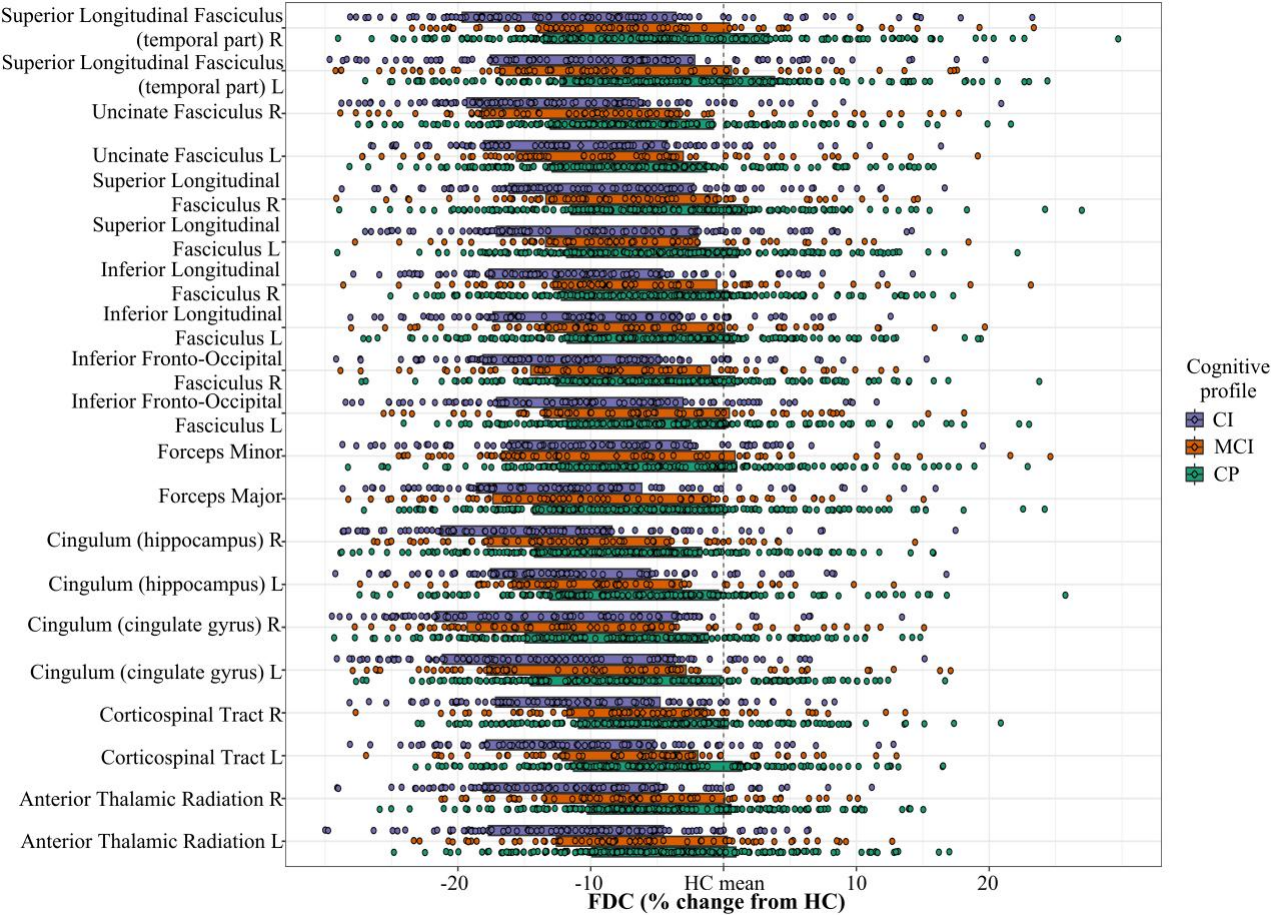
**Significant tracts in MS from baseline FDC tract-of-interest analysis comparing cognitive profiles**. Mean FDC (diamonds) and 95% CIs (bars) within significant tracts (one-way ANOVAs, all *P*-values < 0.001, all *F* > 10.5) are displayed for CI, MCI and CP groups, as a percentage difference from the healthy control mean. FDC, fibre density and cross-section; CIs, confidence intervals; CI, cognitively impaired; MCI, mildly cognitively impaired; CP, cognitively preserved; L, left; R, right.

When comparing the different cognitive profiles (CP, MCI and CI) at baseline, we observed a common pattern for the three measures of a continuously lower integrity from CP to MCI and to CI (Fig. 4 and Supplementary Figs. 11 and 12 for FDC, FD and FC, respectively). Comparisons between pairs of groups in a post-hoc analysis are reported in full in Supplementary Tables 4, 5 and 6 and showed the most extensive microstructural damage within the cingulum, while macrostructural atrophy was most important in bilateral cingulum and the right part of the SLF. As for the previous comparison between clinical phenotypes, discarding white matter lesions involved similar results with a slight increase in effect sizes (Supplementary Figs. 13, 14 and 15). Similarly, when repeating the analyses for patients, results were similar when including treatment information as a cofounding factor.

##### Follow-up

**Figure 5:**
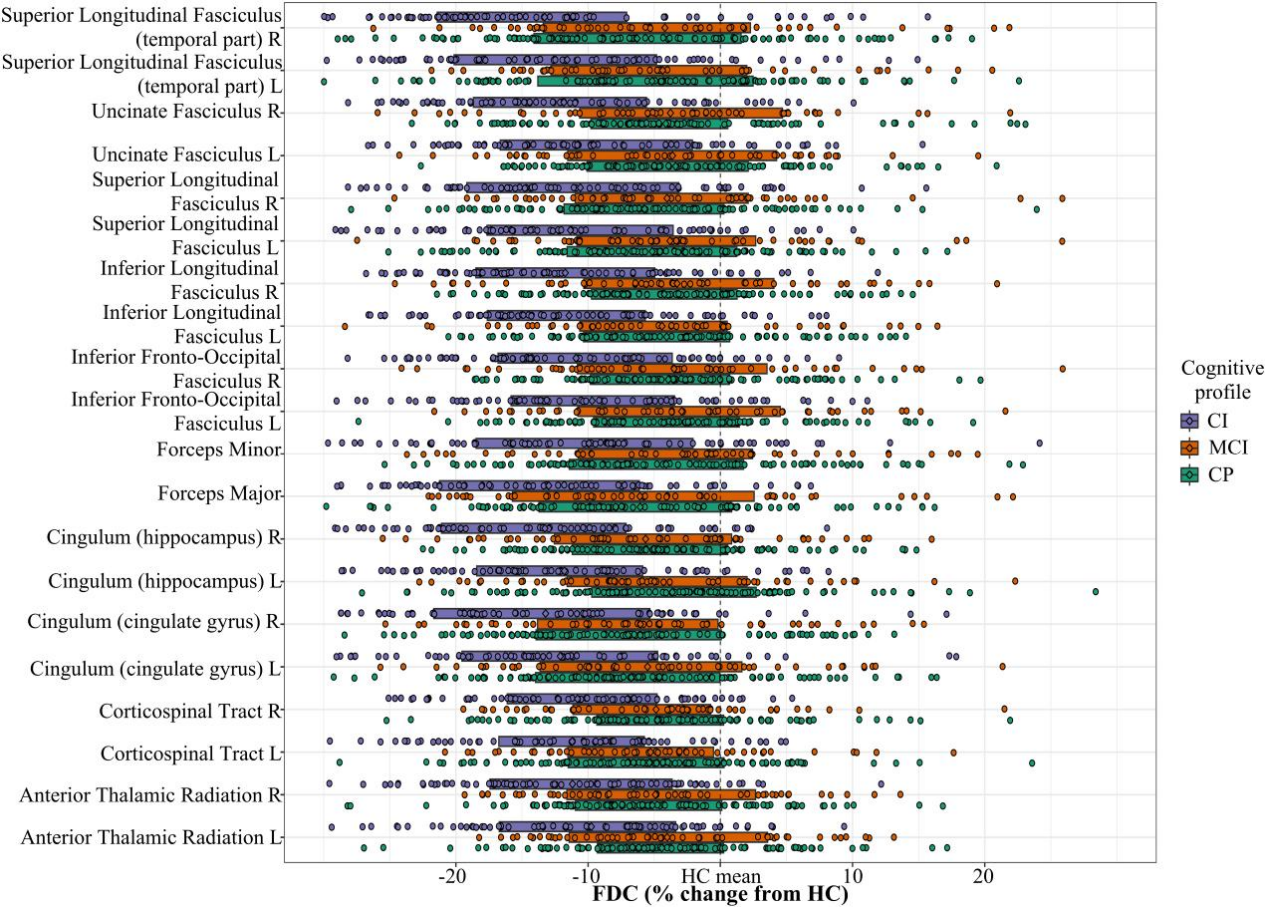
**Significant tracts in MS from follow-up FDC tract-of-interest analysis comparing cognitive profiles**. Mean FDC (diamonds) and 95% CIs (bars) within significant tracts (one-way ANOVAs, all *P*-values < 0.001, all *F* > 13.5) are displayed for CI, MCI and CP groups, as a percentage difference from the healthy control mean. FDC, fibre density and cross-section; CIs, confidence intervals; CI, cognitively impaired; MCI, mildly cognitively impaired; CP, cognitively preserved; L, left; R, right.

Comparable with baseline, worse damage was present in CI compared with both MCI and CP (Fig. 5 and Supplementary Figs. 16 and 17 for FDC, FD and FC, respectively). This was mainly the case for the microstructural damage (FD) and the combined measure FDC. The most extensive damage was present along cingulum and SLF but also within fibres of the corpus callosum. Perhaps due to the reduced sample size, however, CP and MCI had more similar values compared with the baseline visit (Supplementary Tables 7, 8 and 9). Once again, discarding white matter lesions did not lead to different results (Supplementary Figs. 18, 19 and 20). Again, when repeating the analyses for patients, results were similar when including treatment information as a cofounding factor.

### Linear association with clinical disability and cognition

#### Baseline associations

The final cross-sectional linear regression model to find baseline correlates of EDSS (*R*^2^_adjusted_ = 0.36, *P* < 0.001) contained higher age and disease duration, lower deep GM volume and FD of bilateral parahippocampal cingulum (Table 2). Baseline-averaged cognitive performance was most importantly associated (*R*^2^_adjusted_ = 0.38, *P* < 0.001) with male sex and lower educational level, deep and cortical GM volumes, FDC of right IFOF and FC of right SLF (Table 2). Similar results with the same predictors were found when considering fixel-based measures of normal-appearing white matter (data not shown).

**Table 2:** Linear regression analysis for baseline association with clinical disability and cognitive performance

	Model			Predictor		
	Adjusted *R*^2^	*F*	*P*-value	Univariate (*r*)	Multivariate (β)	*P*-value
Baseline average cognitive performance	**0.38**	**16.44**	**<0.001**			
Sex				−0.17	−0.17	**0.008**
Level of education				0.24	0.21	**<0.001**
NDGMV				0.53	0.31	**<0.001**
NCGMV				0.48	0.22	**0.006**
Right IFOF, FDC				0.25	0.52	**<0.001**
Right SLF, FC				0.12	−0.35	**0.008**
Baseline EDSS	**0.36**	**17.35**	**<0.001**			
Age				0.54	0.23	**<0.001**
Disease duration				0.44	0.20	**0.001**
NDGMV				−0.41	−0.27	**<0.001**
Left cingulum (hippocampus), FD				−0.34	−0.19	**0.031**
Right cingulum (hippocampus), FD				−0.32	0.19	**0.032**

Bold values indicate significant findings (*P* < 0.05).

NCGMV, normalized cortical grey matter volume; NDGMV, normalized deep grey matter volume; IFOF, inferior fronto-occipital fasciculus; FDC, fibre density and cross-section; SLF, superior longitudinal fasciculus; FC, fibre cross-section; EDSS, Expanded Disability Status Scale; FD, fibre density.

#### Baseline fixel-based measures predict clinical worsening at follow-up

A 10-fold nested cross-validation approach was used to build a model predictive of EDSS and cognitive decline. We found an optimal and significant (*P* < 0.001) model to predict EDSS at follow-up with the following predictors: age, disease duration, lower cortical GM volume, FD and FC of right parahippocampal cingulum, FD of left parahippocampal cingulum and FDC of left IFOF and right uncinate fasciculus. This model achieved an RMSE = 1.46, *F* = 13.99 and *R*^2^_adj_ = 0.36 (Table 3). When applied to the independent test set, this model achieved an RMSE = 1.37 and *r* = 0.62 for correlations between observed and predicted values. As for average cognitive decline at follow-up, it was significantly (*P* < 0.001) predicted with male sex, lower educational level, deep and cortical GM volumes, FD and FC of forceps major, FDC of right ILF and SLF and FC of right cingulum. This model achieved an RMSE = 0.75, *F* = 14.84 and *R*^2^_adj_ = 0.41 (Table 3). When applied to the independent test set, this model achieved an RMSE = 0.58 and *r* = 0.78 for correlations between observed and predicted values. Similar results with the same predictors were found when considering fixel-based measures of normal-appearing white matter (data not shown).

**Table 3:** Linear regression analysis for predicting follow-up clinical disability and cognitive decline

	Model			Predictor		
	Adjusted *R*^2^	*F*	*P*-value	Univariate (*r*)	Multivariate (β)	*P*-value
Follow-up average cognitive performance	**0.41**	**14.84**	**<0.001**			
Sex				−0.19	−0.21	**0.003**
Level of education				0.22	0.20	**<0.001**
NDGMV				0.53	0.27	**<0.001**
NCGMV				0.52	0.22	**0.008**
Forceps major, FD				0.37	0.20	**0.012**
Right ILF, FDC				0.26	0.08	**0.048**
Right SLF, FDC				0.22	−0.24	**0.034**
Right cingulum (cingulate gyrus), FC				0.10	0.14	**0.041**
Forceps major, FC				0.12	0.18	**0.045**
Follow-up EDSS	**0.36**	**13.99**	**<0.001**			
Age				0.47	0.33	**0.036**
Disease duration				0.39	0.31	**0.029**
NCGMV				−0.50	−0.68	**<0.001**
Left cingulum (hippocampus), FD				−0.30	−0.50	**0.015**
Right cingulum (hippocampus), FD				−0.24	0.63	**0.006**
Right cingulum (hippocampus), FC				−0.11	−0.31	**0.037**
Left IFOF, FDC				−0.17	0.65	**0.002**
Right uncinate fasciculus, FDC				−0.29	−0.58	**0.008**

Bold values indicate significant findings (*P* < 0.05).

NCGMV, normalized cortical grey matter volume; NDGMV, normalized deep grey matter volume; FD, fibre density; ILF, inferior longitudinal fasciculus; FDC, fibre density and cross-section; SLF, superior longitudinal fasciculus; FC, fibre cross-section; EDSS, Expanded Disability Status Scale; IFOF, inferior fronto-occipital fasciculus.

## Discussion

In the present study, we investigated the clinical relevance of white matter degeneration using an advanced fibre-specific DWI model based on fixel-based measures that better accounts for crossing fibres in a large cohort of MS patients. Cross-sectionally, SPMS patients showed more pronounced alterations in FD as well as fibre bundle diameter, while PPMS and RRMS patients displayed a similar profile of WM degeneration. WM damage worsened over time with FD loss mainly seen in progressive patients, with only SPMS subjects showing a more extensive axonal loss at follow-up. Cognitive relevance was highlighted with WM degeneration worsening from CP to MCI and to CI. Fixel-based measures of specific WM pathways were predictive of future cognitive and motor decline over time.

SPMS patients showed the most WM degeneration, followed by PPMS patients which often showed comparable damage with RRMS patients. Longitudinally, however, PPMS showed more changes compared with RRMS. Although some previous studies reported similar imaging characteristics between PPMS and RRMS (in terms of demyelination, number, shape, location and distribution of their lesions),^43,44^ others showed important differences using both conventional and non-conventional imaging.^45-47^ Indeed, PPMS patients are characterized by a faster rate of brain atrophy compared with RRMS,^45^ less focal WM lesions but more prevalent cortical lesions,^46^ elevated creatine and reduced N-acetylaspartate.^47^ Together with our results, this could indicate a stage-specific spreading of damage, with progressive disability in PPMS reflected by an increased gliosis and axonal loss, whereas disability in RRMS is reflected by the cumulative effects of acute inflammatory lesions and axonal loss.

Across all comparisons, FD showed more severe changes compared with FC, indicating more intra-axonal volume loss at the microstructural level. However, a reduction of FC, as a measure of macrostructural WM atrophy, was only detected in SPMS patients. In recent studies, authors also showed greater reductions of FD compared with FC in patients with optic neuritis along the optic radiation.^20^ Others showed more important changes in FC in progressive patients compared with RRMS.^48^ These findings suggest that a reduction of FD might prevail over measurable changes seen in FC in the early stages, whereas changes in FC might become more pronounced later in the disease, leading to irreversible large-scale axonal loss. As such, predicting clinical progression in MS might require disease stage-specific measures, using microscopic changes in early disease and both micro- and macro-scale measurements in progressive MS.

A substantial loss was seen in MS for FD and volume within specific white matter structures. Damage only partially overlapped with lesion probability maps. This result is consistent with recent findings showing altered fixel-based measures along with little lesion activity.^21,49^ This could indicate that axonal loss within these tracts is more likely caused by trans-synaptic degeneration due to lesions in second-order tracts and highlights the sensitivity of such a method to quantify damage in normal-appearing WM.

Affected tracts were not the same for cognitive compared with motor impairment. For cognition, long association fibres such as the cingulum bundle, the uncinate fasciculus and inferior and superior longitudinal fasciculus were especially important. Although some of these tracts have been previously reported, only DTI models were used and they could not measure damage within these specific bundles but more likely the effect of crossing fibres. Interestingly, these anatomical pathways connect default-mode network (DMN) brain regions that have previously been described as functionally implicated in MS and particularly in CI.^50,51^ For example, the cingulum bundle form connections between the anterior medial prefrontal cortex, posterior cingulate cortex and medial temporal lobe.^52^ The uncinate fasciculus is connected to both the ventral medial prefrontal cortex and the hippocampus.^53^ These results could explain the preferential sensitivity of the DMN during the disease course, as structural disconnection could induce atrophy as well as hyperconnectivity.^54^ We found a strong association between baseline damage to higher order pathways, such as forceps major, ILF, SLF and cingulum, and future cognitive decline in MS patients. This is in line with previous reports of the involvement of these tracts in CI in MS.^8,55,56^ Additionally, alterations to both micro- and macrostructure at baseline of the cingulum, IFOF and uncinate fasciculus were predictors of future physical disability after 5 years of disease evolution. These pathways are congruent with the regions of white matter abnormalities recently reported to be associated with EDSS using both DTI^57^ and structural connectivity.^58^

While some fixel-based results in this study overlap to some extent with regions identified in standard DTI findings, the fixel-based measures offer much greater anatomical specificity and valuable insights into the likely nature of these abnormalities by identifying tract-specific changes and accounting for WM atrophy that occur along the disease.^17^ Furthermore, the previously depicted altered pathways were mostly long association fibres connecting distant brain regions, which cross with other fibre pathways at many points along their length, so classical DTI model cannot reliably estimate the orientation of underlying fibres.^17^ This selective vulnerability of these long-range fibres may be related to their locations close to the ventricles, where WM lesions are predominant. Another hypothesis could be that the higher metabolic level of long-range connections makes them more vulnerable to oxidative stress and therefore to energy failure under stress.^59^

Despite these technical advantages, this study had some limitations that need to be addressed in future works. First, our diffusion acquisition only included 30 directions with a *b*-value of 1000 s/mm^2^. While it is recommended to use 45 directions with a higher *b*-value, several studies using a similar protocol still yielded promising results, demonstrating the feasibility of FBA with more conventional acquisition schemes which could therefore reduce acquisition times.^60^ Additionally, we controlled the different fixels generated within our pipeline and did not detect any incoherent directionality or response function. However, we should be careful with the interpretation of the FD measure, as it is approximately proportional to the total amount of intra-cellular volume of axons and is more specific at high *b*-values.^17^ Future studies acquiring high angular resolution diffusion imaging are required to validate our results. In addition, while the improved specificity of FBA relative to other voxel-based approaches has been well investigated,^60^ alternative multi-compartment methods, such as neurite orientation dispersion and density imaging (NODDI),^61^ have been introduced to quantify WM microstructural properties with greater specificity to intra-cellular properties and separate these from effects due to the extra-cellular compartment. Unfortunately, our diffusion data did not meet the requirements for such an analysis, requiring the acquisition of multiple *b*-values. On the other hand, even though NODDI improves the specificity of WM microstructure, it is inherently unable to assign significant effects to specific fibre populations, and neurite density is not separately quantified for crossing neurite populations.^60^ Another important consideration is the absence of very early MS patients. Indeed, our population had ∼15 years of disease duration, with shortest disease durations at around 6 years after diagnosis. So, although this is a longitudinal study, we cannot infer the type and trajectory of damage in the earliest stages of the disease. Finally, our interpretation of FD loss as axonal loss was based on solid mathematical models,^17^ but histological validation of these fixel-based measures using post-mortem MRI may help us interpret our results and better understand the underlying pathology.

In summary, we have used fixel-based measures to investigate the evolution and clinical relevance of tract-specific degeneration in MS patients. Progressive MS showed the fastest and most extensive spreading of white matter degeneration, even when lesioned areas were excluded. Tracts in different major cortical functional networks were affected between cognitively and physically impaired patients related to these respective symptoms. White matter degeneration was predictive of both future cognitive decline and clinical disability, suggesting that these measures could serve as potential markers with higher specificity of axonal degeneration in monitoring treatment efficacy and predicting clinical outcomes.

## Supplementary Material

fcae018_Supplementary_Data

## Data Availability

The data that supported the findings of this study are available from the corresponding author upon reasonable request.
