# Supplementary material for: Longitudinal fibre-specific white matter damage predicts cognitive decline in multiple sclerosis
Source: Brain Commun. 2024 Jan 27;6(1):fcae018. doi: 10.1093/braincomms/fcae018 (PMC10853982; doi:10.1093/braincomms/fcae018)

Supplementary material

Supplementary Table 1. Post-hoc comparisons between clinical phenotypes for FD at baseline

Tract of interest			P-value
**Anterior thalamic radiation L**	HC	RRMS	**< 0.001**
		SPMS	**< 0.001**
		PPMS	0.08
	RRMS	SPMS	**0.002**
		PPMS	1
	SPMS	PPMS	**0.001**
**Anterior thalamic radiation R**	HC	RRMS	**< 0.001**
		SPMS	**< 0.001**
		PPMS	0.058
	RRMS	SPMS	**0.03**
		PPMS	1
	SPMS	PPMS	**0.019**
**Cingulum (cingulate gyrus) L**	HC	RRMS	**< 0.001**
		SPMS	**< 0.001**
		PPMS	0.25
	RRMS	SPMS	1
		PPMS	0.14
	SPMS	PPMS	**0.016**
**Cingulum (cingulate gyrus) R**	HC	RRMS	**< 0.001**
		SPMS	**< 0.001**
		PPMS	0.27
	RRMS	SPMS	0.85
		PPMS	0.21
	SPMS	PPMS	**0.019**
**Cingulum (hippocampus) L**	HC	RRMS	**< 0.001**
		SPMS	**< 0.001**
		PPMS	**0.003**
	RRMS	SPMS	0.08
		PPMS	1
	SPMS	PPMS	**0.034**
**Cingulum (hippocampus) R**	HC	RRMS	**< 0.001**
		SPMS	**< 0.001**
		PPMS	**0.001**
	RRMS	SPMS	1
		PPMS	0.94
	SPMS	PPMS	0.26
**Forceps major**	HC	RRMS	**< 0.001**
		SPMS	**< 0.001**
		PPMS	0.17
	RRMS	SPMS	0.13
		PPMS	0.93
	SPMS	PPMS	**0.019**
**Forceps minor**	HC	RRMS	**< 0.001**
		SPMS	**< 0.001**
		PPMS	**0.024**
	RRMS	SPMS	1
		PPMS	1
	SPMS	PPMS	1

Supplementary Table 1. Post-hoc comparisons between clinical phenotypes for FD at baseline (next)

**Tract of interest**			**P-value**
**IFOF L**	HC	RRMS	< **0.001**
		SPMS	**0.001**
		PPMS	1
	RRMS	SPMS	1
		PPMS	0.33
	SPMS	PPMS	0.16
**IFOF R**	HC	RRMS	< **0.001**
		SPMS	< **0.001**
		PPMS	1
	RRMS	SPMS	0.35
		PPMS	0.47
	SPMS	PPMS	**0.019**
**ILF L**	HC	RRMS	< **0.001**
		SPMS	**0.003**
		PPMS	0.16
	RRMS	SPMS	1
		PPMS	1
	SPMS	PPMS	1
**ILF R**	HC	RRMS	< **0.001**
		SPMS	< **0.001**
		PPMS	0.09
	RRMS	SPMS	1
		PPMS	1
	SPMS	PPMS	1
**SLF L**	HC	RRMS	< **0.001**
		SPMS	< **0.001**
		PPMS	0.31
	RRMS	SPMS	0.35
		PPMS	1
	SPMS	PPMS	0.10
**SLF R**	HC	RRMS	< **0.001**
		SPMS	< **0.001**
		PPMS	0.27
	RRMS	SPMS	0.13
		PPMS	1
	SPMS	PPMS	0.051
**Uncinate fasciculus L**	HC	RRMS	< **0.001**
		SPMS	< **0.001**
		PPMS	**0.036**
	RRMS	SPMS	1
		PPMS	1
	SPMS	PPMS	0.32
**Uncinate fasciculus R**	HC	RRMS	< **0.001**
		SPMS	< **0.001**
		PPMS	**0.021**
	RRMS	SPMS	1
		PPMS	1
	SPMS	PPMS	1

FD: Fiber density; HC: healthy controls; RRMS: relapsing-remitting multiple sclerosis; SPMS: secondary-progressive MS; PPMS: primary-progressive MS; L: left; R: right; IFOF: inferior fronto-occipital fasciculus; ILF: inferior longitudinal fasciculus; SLF: superior longitudinal fasciculus. All p-values are Bonferroni corrected and a p-value < 0.05 is considered significant.

Supplementary Table 2. Post-hoc comparisons between clinical phenotypes for FC at baseline

Tract of interest			P-value
**Corticospinal tract L**	HC	RRMS	**< 0.001**
		SPMS	**< 0.001**
		PPMS	**0.006**
	RRMS	SPMS	**0.046**
		PPMS	1
	SPMS	PPMS	0.10
**Corticospinal tract R**	HC	RRMS	**< 0.001**
		SPMS	**< 0.001**
		PPMS	**0.013**
	RRMS	SPMS	**0.041**
		PPMS	1
	SPMS	PPMS	0.050
**Cingulum (cingulate gyrus) L**	HC	RRMS	**< 0.001**
		SPMS	**< 0.001**
		PPMS	**0.009**
	RRMS	SPMS	0.069
		PPMS	1
	SPMS	PPMS	0.41
**Cingulum (cingulate gyrus) R**	HC	RRMS	**< 0.001**
		SPMS	**< 0.001**
		PPMS	**0.001**
	RRMS	SPMS	0.16
		PPMS	1
	SPMS	PPMS	0.92
**IFOF L**	HC	RRMS	**< 0.001**
		SPMS	**0.002**
		PPMS	1
	RRMS	SPMS	1
		PPMS	0.4
	SPMS	PPMS	0.47
**IFOF R**	HC	RRMS	**< 0.001**
		SPMS	**< 0.001**
		PPMS	0.097
	RRMS	SPMS	1
		PPMS	1
	SPMS	PPMS	0.76
**ILF L**	HC	RRMS	**< 0.001**
		SPMS	**0.006**
		PPMS	0.10
	RRMS	SPMS	1
		PPMS	1
	SPMS	PPMS	1
**ILF R**	HC	RRMS	**< 0.001**
		SPMS	**0.002**
		PPMS	**0.049**
	RRMS	SPMS	1
		PPMS	1
	SPMS	PPMS	1

Supplementary Table 2. Post-hoc comparisons between clinical phenotypes for FC at baseline (next)

**Tract of interest**			**P-value**
**SLF L**	HC	RRMS	**< 0.001**
		SPMS	**0.007**
		PPMS	0.42
	RRMS	SPMS	1
		PPMS	1
	SPMS	PPMS	1
**SLF R**	HC	RRMS	**< 0.001**
		SPMS	**0.011**
		PPMS	1
	RRMS	SPMS	1
		PPMS	1
	SPMS	PPMS	1
**Uncinate fasciculus L**	HC	RRMS	**< 0.001**
		SPMS	0.12
		PPMS	0.25
	RRMS	SPMS	1
		PPMS	1
	SPMS	PPMS	1
**Uncinate fasciculus R**	HC	RRMS	**< 0.001**
		SPMS	**0.004**
		PPMS	**0.040**
	RRMS	SPMS	1
		PPMS	1
	SPMS	PPMS	1
**SLF (temporal part) R**	HC	RRMS	**0.002**
		SPMS	**0.037**
		PPMS	**0.008**
	RRMS	SPMS	1
		PPMS	1
	SPMS	PPMS	1

FC: Fiber cross-section; HC: healthy controls; RRMS: relapsing-remitting multiple sclerosis; SPMS: secondary-progressive MS; PPMS: primary-progressive MS; L: left; R: right; IFOF: inferior fronto-occipital fasciculus; ILF: inferior longitudinal fasciculus; SLF: superior longitudinal fasciculus. All p-values are Bonferroni corrected and a p-value < 0.05 is considered significant.

Supplementary Table 3. Post-hoc comparisons between clinical phenotypes for FDC at baseline

**Tract of interest**			**P-value**
**Anterior thalamic radiation L**	HC	RRMS	**< 0.001**
		SPMS	**< 0.001**
		PPMS	**0.022**
	RRMS	SPMS	0.22
		PPMS	1
	SPMS	PPMS	0.086
**Anterior thalamic radiation R**	HC	RRMS	**< 0.001**
		SPMS	**< 0.001**
		PPMS	**0.007**
	RRMS	SPMS	0.44
		PPMS	1
	SPMS	PPMS	0.23
**Corticospinal tract L**	HC	RRMS	**< 0.001**
		SPMS	**< 0.001**
		PPMS	0.15
	RRMS	SPMS	0.35
		PPMS	0.54
	SPMS	PPMS	**0.023**
**Corticospinal tract R**	HC	RRMS	**< 0.001**
		SPMS	**< 0.001**
		PPMS	0.10
	RRMS	SPMS	0.16
		PPMS	0.98
	SPMS	PPMS	**0.024**
**Cingulum (cingulate gyrus) L**	HC	RRMS	**< 0.001**
		SPMS	**< 0.001**
		PPMS	**0.006**
	RRMS	SPMS	0.25
		PPMS	1
	SPMS	PPMS	**0.046**
**Cingulum (cingulate gyrus) R**	HC	RRMS	**< 0.001**
		SPMS	**< 0.001**
		PPMS	**0.001**
	RRMS	SPMS	0.29
		PPMS	1
	SPMS	PPMS	0.076
**Cingulum (hippocampus) L**	HC	RRMS	**< 0.001**
		SPMS	**< 0.001**
		PPMS	**< 0.001**
	RRMS	SPMS	0.37
		PPMS	1
	SPMS	PPMS	1
**Cingulum (hippocampus) R**	HC	RRMS	**< 0.001**
		SPMS	**< 0.001**
		PPMS	**< 0.001**
	RRMS	SPMS	1
		PPMS	1
	SPMS	PPMS	1

Supplementary Table 3. Post-hoc comparisons between clinical phenotypes for FDC at baseline (next)

Tract of interest			P-value
**Forceps major**	HC	RRMS	< **0.001**
		SPMS	< **0.001**
		PPMS	0.10
	RRMS	SPMS	0.18
		PPMS	0.83
	SPMS	PPMS	**0.021**
**Forceps minor**	HC	RRMS	**< 0.001**
		SPMS	**0.001**
		PPMS	**< 0.001**
	RRMS	SPMS	1
		PPMS	1
	SPMS	PPMS	1
**IFOF L**	HC	RRMS	**< 0.001**
		SPMS	< **0.001**
		PPMS	0.56
	RRMS	SPMS	1
		PPMS	0.22
	SPMS	PPMS	0.17
**IFOF R**	HC	RRMS	**< 0.001**
		SPMS	< **0.001**
		PPMS	0.12
	RRMS	SPMS	1
		PPMS	0.61
	SPMS	PPMS	0.097
**ILF L**	HC	RRMS	**< 0.001**
		SPMS	< **0.001**
		PPMS	**0.018**
	RRMS	SPMS	1
		PPMS	1
	SPMS	PPMS	1
**ILF R**	HC	RRMS	< **0.001**
		SPMS	< **0.001**
		PPMS	**0.010**
	RRMS	SPMS	1
		PPMS	1
	SPMS	PPMS	1
**SLF L**	HC	RRMS	**< 0.001**
		SPMS	**< 0.001**
		PPMS	0.14
	RRMS	SPMS	1
		PPMS	1
	SPMS	PPMS	0.36
**SLF R**	HC	RRMS	**< 0.001**
		SPMS	**< 0.001**
		PPMS	0.26
	RRMS	SPMS	0.81
		PPMS	1
	SPMS	PPMS	0.15

Supplementary Table 3. Post-hoc comparisons between clinical phenotypes for FDC at baseline (next)

**Tract of interest**			**P-value**
**Uncinate fasciculus L**	HC	RRMS	**< 0.001**
		SPMS	**< 0.001**
		PPMS	**0.017**
	RRMS	SPMS	1
		PPMS	1
	SPMS	PPMS	1
**Uncinate fasciculus R**	HC	RRMS	**< 0.001**
		SPMS	**< 0.001**
		PPMS	**0.002**
	RRMS	SPMS	1
		PPMS	1
	SPMS	PPMS	1
**SLF (temporal part) L**	HC	RRMS	**< 0.001**
		SPMS	**0.016**
		PPMS	0.36
	RRMS	SPMS	1
		PPMS	1
	SPMS	PPMS	1
**SLF (temporal part) R**	HC	RRMS	**0.001**
		SPMS	**0.001**
		PPMS	**0.022**
	RRMS	SPMS	1
		PPMS	1
	SPMS	PPMS	1

FDC: Fiber density and cross-section; HC: healthy controls; RRMS: relapsing-remitting multiple sclerosis; SPMS: secondary-progressive MS; PPMS: primary-progressive MS; L: left; R: right; IFOF: inferior fronto-occipital fasciculus; ILF: inferior longitudinal fasciculus; SLF: superior longitudinal fasciculus. All p-values are Bonferroni corrected and a p-value < 0.05 is considered significant.

Supplementary Table 4. Post-hoc comparisons between cognitive profiles for FD at baseline

Tract of interest			P-value
**Anterior thalamic radiation L**	HC	CP	< **0.001**
		MCI	< **0.001**
		CI	< **0.001**
	CP	MCI	1
		CI	< **0.001**
	MCI	CI	< **0.001**
**Anterior thalamic radiation R**	HC	CP	< **0.001**
		MCI	< **0.001**
		CI	< **0.001**
	CP	MCI	1
		CI	< **0.001**
	MCI	CI	< **0.001**
**Cingulum (cingulate gyrus) L**	HC	CP	< **0.001**
		MCI	< **0.001**
		CI	< **0.001**
	CP	MCI	0.77
		CI	< **0.001**
	MCI	CI	**0.006**
**Cingulum (cingulate gyrus) R**	HC	CP	< **0.001**
		MCI	< **0.001**
		CI	< **0.001**
	CP	MCI	0.35
		CI	< **0.001**
	MCI	CI	0.13
**Cingulum (hippocampus) L**	HC	CP	< **0.001**
		MCI	< **0.001**
		CI	< **0.001**
	CP	MCI	**0.049**
		CI	< **0.001**
	MCI	CI	0.11
**Cingulum (hippocampus) R**	HC	CP	< **0.001**
		MCI	< **0.001**
		CI	< **0.001**
	CP	MCI	0.18
		CI	< **0.001**
	MCI	CI	**0.032**
**Forceps major**	HC	CP	< **0.001**
		MCI	< **0.001**
		CI	< **0.001**
	CP	MCI	0.83
		CI	< **0.001**
	MCI	CI	**0.001**
**Forceps minor**	HC	CP	**0.003**
		MCI	< **0.001**
		CI	< **0.001**
	CP	MCI	0.25
		CI	< **0.001**
	MCI	CI	0.62

Supplementary Table 4. Post-hoc comparisons between cognitive profiles for FD at baseline (next)

Tract of interest			P-value
**IFOF L**	HC	CP	**0.009**
		MCI	0.057
		CI	**< 0.001**
	CP	MCI	1
		CI	**0.002**
	MCI	CI	**0.029**
**IFOF R**	HC	CP	**0.042**
		MCI	**0.014**
		CI	**< 0.001**
	CP	MCI	1
		CI	**< 0.001**
	MCI	CI	0.062
**ILF L**	HC	CP	**0.004**
		MCI	**0.015**
		CI	**< 0.001**
	CP	MCI	1
		CI	**0.006**
	MCI	CI	0.13
**ILF R**	HC	CP	**0.001**
		MCI	**0.005**
		CI	**< 0.001**
	CP	MCI	1
		CI	**0.001**
	MCI	CI	**0.048**
**SLF L**	HC	CP	**0.001**
		MCI	**0.001**
		CI	**< 0.001**
	CP	MCI	1
		CI	**< 0.001**
	MCI	CI	0.071
**SLF R**	HC	CP	**0.001**
		MCI	**0.001**
		CI	**< 0.001**
	CP	MCI	1
		CI	**< 0.001**
	MCI	CI	0.059
**Uncinate fasciculus L**	HC	CP	**< 0.001**
		MCI	**< 0.001**
		CI	**< 0.001**
	CP	MCI	1
		CI	**0.002**
	MCI	CI	0.23
**Uncinate fasciculus R**	HC	CP	**< 0.001**
		MCI	**< 0.001**
		CI	**< 0.001**
	CP	MCI	0.47
		CI	**< 0.001**
	MCI	CI	0.59

Supplementary Table 4. Post-hoc comparisons between cognitive profiles for FD at baseline (next)

**Tract of interest**			**P-value**
**SLF (temporal part) R**	HC	CP	0.95
		MCI	0.80
		CI	**< 0.001**
	CP	MCI	1
		CI	**0.008**
	MCI	CI	0.16

FD: Fiber density; HC: healthy controls; CP: cognitively preserved; MCI: mildly cognitively impaired; CI: cognitively impaired; L: left; R: right; IFOF: inferior fronto-occipital fasciculus; ILF: inferior longitudinal fasciculus; SLF: superior longitudinal fasciculus. All p-values are Bonferroni corrected and a p-value < 0.05 is considered significant.

Supplementary Table 5. Post-hoc comparisons between cognitive profiles for FC at baseline

**Tract of interest**			**P-value**
**Corticospinal tract L**	HC	CP	**< 0.001**
		MCI	**< 0.001**
		CI	**< 0.001**
	CP	MCI	0.61
		CI	**< 0.001**
	MCI	CI	**< 0.001**
**Corticospinal tract R**	HC	CP	**< 0.001**
		MCI	**< 0.001**
		CI	**< 0.001**
	CP	MCI	0.99
		CI	**< 0.001**
	MCI	CI	**< 0.001**
**Cingulum (cingulate gyrus) L**	HC	CP	**< 0.001**
		MCI	**0.001**
		CI	**< 0.001**
	CP	MCI	1
		CI	**0.003**
	MCI	CI	0.072
**Cingulum (cingulate gyrus) R**	HC	CP	**< 0.001**
		MCI	**< 0.001**
		CI	**< 0.001**
	CP	MCI	1
		CI	**< 0.001**
	MCI	CI	**0.014**
**IFOF L**	HC	CP	**0.027**
		MCI	**0.001**
		CI	**< 0.001**
	CP	MCI	0.51
		CI	**< 0.001**
	MCI	CI	**0.041**
**IFOF R**	HC	CP	**0.001**
		MCI	**< 0.001**
		CI	**< 0.001**
	CP	MCI	0.18
		CI	**< 0.001**
	MCI	CI	**0.015**
**ILF L**	HC	CP	**0.004**
		MCI	**0.005**
		CI	**< 0.001**
	CP	MCI	1
		CI	**0.001**
	MCI	CI	0.074
**ILF R**	HC	CP	**0.019**
		MCI	**0.010**
		CI	**< 0.001**
	CP	MCI	1
		CI	**< 0.001**
	MCI	CI	**0.032**

Supplementary Table 5. Post-hoc comparisons between cognitive profiles for FC at baseline (next)

**Tract of interest**			**P-value**
**SLF L**	HC	CP	**0.015**
		MCI	**0.017**
		CI	**< 0.001**
	CP	MCI	1
		CI	**< 0.001**
	MCI	CI	**0.048**
**SLF R**	HC	CP	**0.010**
		MCI	0.074
		CI	**< 0.001**
	CP	MCI	1
		CI	**0.035**
	MCI	CI	0.17
**Uncinate fasciculus L**	HC	CP	**0.009**
		MCI	**0.006**
		CI	**< 0.001**
	CP	MCI	1
		CI	**0.002**
	MCI	CI	0.23
**Uncinate fasciculus R**	HC	CP	**0.003**
		MCI	**< 0.001**
		CI	**< 0.001**
	CP	MCI	0.98
		CI	**< 0.001**
	MCI	CI	0.12
**SLF (temporal part) R**	HC	CP	0.089
		MCI	**0.03**
		CI	**< 0.001**
	CP	MCI	1
		CI	**0.002**
	MCI	CI	0.26

FC: Fiber cross-section; HC: healthy controls; CP: cognitively preserved; MCI: mildly cognitively impaired; CI: cognitively impaired; L: left; R: right; IFOF: inferior fronto-occipital fasciculus; ILF: inferior longitudinal fasciculus; SLF: superior longitudinal fasciculus. All p-values are Bonferroni corrected and a p-value < 0.05 is considered significant.

Supplementary Table 6. Post-hoc comparisons between cognitive profiles for FDC at baseline

**Tract of interest**			**P-value**
**Anterior thalamic radiation L**	HC	CP	**< 0.001**
		MCI	**< 0.001**
		CI	**< 0.001**
	CP	MCI	0.54
		CI	**< 0.001**
	MCI	CI	**< 0.001**
**Anterior thalamic radiation R**	HC	CP	**< 0.001**
		MCI	**< 0.001**
		CI	**< 0.001**
	CP	MCI	0.43
		CI	**< 0.001**
	MCI	CI	**< 0.001**
**Corticospinal tract L**	HC	CP	**< 0.001**
		MCI	**< 0.001**
		CI	**< 0.001**
	CP	MCI	1
		CI	**< 0.001**
	MCI	CI	**< 0.001**
**Corticospinal tract R**	HC	CP	**< 0.001**
		MCI	**< 0.001**
		CI	**< 0.001**
	CP	MCI	1
		CI	**< 0.001**
	MCI	CI	**< 0.001**
**Cingulum (cingulate gyrus) L**	HC	CP	**< 0.001**
		MCI	**< 0.001**
		CI	**< 0.001**
	CP	MCI	1
		CI	**< 0.001**
	MCI	CI	**0.005**
**Cingulum (cingulate gyrus) R**	HC	CP	**< 0.001**
		MCI	**< 0.001**
		CI	**< 0.001**
	CP	MCI	0.62
		CI	**< 0.001**
	MCI	CI	**0.009**
**Cingulum (hippocampus) L**	HC	CP	**< 0.001**
		MCI	**< 0.001**
		CI	**< 0.001**
	CP	MCI	0.071
		CI	**< 0.001**
	MCI	CI	0.77
**Cingulum (hippocampus) R**	HC	CP	**< 0.001**
		MCI	**< 0.001**
		CI	**< 0.001**
	CP	MCI	0.23
		CI	**< 0.001**
	MCI	CI	0.19

Supplementary Table 6. Post-hoc comparisons between cognitive profiles for FDC at baseline (next)

**Tract of interest**			**P-value**
**Forceps major**	HC	CP	**< 0.001**
		MCI	**< 0.001**
		CI	**< 0.001**
	CP	MCI	0.16
		CI	**< 0.001**
	MCI	CI	**0.012**
**Forceps minor**	HC	CP	**0.001**
		MCI	**< 0.001**
		CI	**< 0.001**
	CP	MCI	0.45
		CI	**< 0.001**
	MCI	CI	0.50
**IFOF L**	HC	CP	**< 0.001**
		MCI	**< 0.001**
		CI	**< 0.001**
	CP	MCI	1
		CI	**< 0.001**
	MCI	CI	**0.01**
**IFOF R**	HC	CP	**< 0.001**
		MCI	**< 0.001**
		CI	**< 0.001**
	CP	MCI	0.42
		CI	**< 0.001**
	MCI	CI	**0.007**
**ILF L**	HC	CP	**< 0.001**
		MCI	**< 0.001**
		CI	**< 0.001**
	CP	MCI	1
		CI	**< 0.001**
	MCI	CI	**0.025**
**ILF R**	HC	CP	**< 0.001**
		MCI	**< 0.001**
		CI	**< 0.001**
	CP	MCI	1
		CI	**< 0.001**
	MCI	CI	**0.019**
**SLF L**	HC	CP	**0.001**
		MCI	**< 0.001**
		CI	**< 0.001**
	CP	MCI	1
		CI	**< 0.001**
	MCI	CI	**0.041**
**SLF R**	HC	CP	**< 0.001**
		MCI	**0.001**
		CI	**< 0.001**
	CP	MCI	1
		CI	**< 0.001**
	MCI	CI	0.062

Supplementary Table 6. Post-hoc comparisons between cognitive profiles for FDC at baseline (next)

**Tract of interest**			**P-value**
**Uncinate fasciculus L**	HC	CP	**< 0.001**
		MCI	**< 0.001**
		CI	**< 0.001**
	CP	MCI	1
		CI	**< 0.001**
	MCI	CI	0.11
**Uncinate fasciculus R**	HC	CP	**< 0.001**
		MCI	**< 0.001**
		CI	**< 0.001**
	CP	MCI	0.66
		CI	**< 0.001**
	MCI	CI	0.088
**SLF (temporal part) L**	HC	CP	**0.032**
		MCI	**0.001**
		CI	**< 0.001**
	CP	MCI	0.45
		CI	**0.005**
	MCI	CI	1
**SLF (temporal part) R**	HC	CP	0.059
		MCI	**0.009**
		CI	**< 0.001**
	CP	MCI	1
		CI	**< 0.001**
	MCI	CI	0.16

FDC: Fiber density and cross-section; HC: healthy controls; CP: cognitively preserved; MCI: mildly cognitively impaired; CI: cognitively impaired; L: left; R: right; IFOF: inferior fronto-occipital fasciculus; ILF: inferior longitudinal fasciculus; SLF: superior longitudinal fasciculus. All p-values are Bonferroni corrected and a p-value < 0.05 is considered significant.

Supplementary Table 7. Post-hoc comparisons between cognitive profiles for FD at follow-up

**Tract of interest**			**P-value**
**Anterior thalamic radiation L**	HC	CP	**0.033**
		MCI	0.060
		CI	**< 0.001**
	CP	MCI	1
		CI	**< 0.001**
	MCI	CI	**< 0.001**
**Anterior thalamic radiation R**	HC	CP	**0.026**
		MCI	**0.012**
		CI	**< 0.001**
	CP	MCI	1
		CI	**< 0.001**
	MCI	CI	**< 0.001**
**Cingulum (cingulate gyrus) L**	HC	CP	**< 0.001**
		MCI	**0.001**
		CI	**< 0.001**
	CP	MCI	1
		CI	**< 0.001**
	MCI	CI	**< 0.001**
**Cingulum (cingulate gyrus) R**	HC	CP	**0.001**
		MCI	**0.002**
		CI	**< 0.001**
	CP	MCI	1
		CI	**< 0.001**
	MCI	CI	**< 0.001**
**Cingulum (hippocampus) L**	HC	CP	0.057
		MCI	**0.003**
		CI	**< 0.001**
	CP	MCI	0.91
		CI	**< 0.001**
	MCI	CI	**< 0.001**
**Cingulum (hippocampus) R**	HC	CP	**0.002**
		MCI	**< 0.001**
		CI	**< 0.001**
	CP	MCI	1
		CI	**< 0.001**
	MCI	CI	**< 0.001**
**Forceps major**	HC	CP	**0.033**
		MCI	**0.005**
		CI	**< 0.001**
	CP	MCI	1
		CI	**< 0.001**
	MCI	CI	**< 0.001**
**Forceps minor**	HC	CP	**< 0.001**
		MCI	**0.006**
		CI	**< 0.001**
	CP	MCI	1
		CI	**< 0.001**
	MCI	CI	**0.003**

Supplementary Table 7. Post-hoc comparisons between cognitive profiles for FD at follow-up (next)

Tract of interest			P-value
**IFOF L**	HC	CP	0.33
		MCI	0.54
		CI	**< 0.001**
	CP	MCI	1
		CI	**< 0.001**
	MCI	CI	**0.002**
**IFOF R**	HC	CP	0.37
		MCI	0.20
		CI	**< 0.001**
	CP	MCI	1
		CI	**< 0.001**
	MCI	CI	**0.001**
**ILF L**	HC	CP	0.10
		MCI	0.074
		CI	**< 0.001**
	CP	MCI	1
		CI	**< 0.001**
	MCI	CI	**0.001**
**ILF R**	HC	CP	**0.049**
		MCI	**0.019**
		CI	**< 0.001**
	CP	MCI	1
		CI	**< 0.001**
	MCI	CI	**0.002**
**SLF L**	HC	CP	**0.016**
		MCI	**0.034**
		CI	**< 0.001**
	CP	MCI	1
		CI	**< 0.001**
	MCI	CI	**0.001**
**SLF R**	HC	CP	**0.002**
		MCI	**0.004**
		CI	**< 0.001**
	CP	MCI	1
		CI	**< 0.001**
	MCI	CI	**0.004**
**Uncinate fasciculus L**	HC	CP	**0.002**
		MCI	**0.013**
		CI	**< 0.001**
	CP	MCI	1
		CI	**< 0.001**
	MCI	CI	**0.002**
**Uncinate fasciculus R**	HC	CP	**0.005**
		MCI	**0.002**
		CI	**< 0.001**
	CP	MCI	1
		CI	**0.001**
	MCI	CI	**0.047**

Supplementary Table 7. Post-hoc comparisons between cognitive profiles for FD at follow-up (next)

Tract of interest			P-value
**SLF (temporal part) R**	HC	CP	0.060
		MCI	0.24
		CI	**< 0.001**
	CP	MCI	1
		CI	0.10
	MCI	CI	0.16

FD: Fiber density; HC: healthy controls; CP: cognitively preserved; MCI: mildly cognitively impaired; CI: cognitively impaired; L: left; R: right; IFOF: inferior fronto-occipital fasciculus; ILF: inferior longitudinal fasciculus; SLF: superior longitudinal fasciculus. All p-values are Bonferroni corrected and a p-value < 0.05 is considered significant.

Supplementary Table 8. Post-hoc comparisons between cognitive profiles for FC at follow-up

Tract of interest			P-value
**Corticospinal tract L**	HC	CP	**0.002**
		MCI	**< 0.001**
		CI	**< 0.001**
	CP	MCI	1
		CI	**< 0.001**
	MCI	CI	**< 0.001**
**Corticospinal tract R**	HC	CP	**0.028**
		MCI	**0.008**
		CI	**< 0.001**
	CP	MCI	1
		CI	**< 0.001**
	MCI	CI	**< 0.001**
**Cingulum (cingulate gyrus) L**	HC	CP	0.97
		MCI	0.33
		CI	**< 0.001**
	CP	MCI	1
		CI	**< 0.001**
	MCI	CI	**0.017**
**Cingulum (cingulate gyrus) R**	HC	CP	0.64
		MCI	0.055
		CI	**< 0.001**
	CP	MCI	1
		CI	**< 0.001**
	MCI	CI	**0.016**
**IFOF L**	HC	CP	1
		MCI	0.73
		CI	**< 0.001**
	CP	MCI	1
		CI	**< 0.001**
	MCI	CI	**0.001**
**IFOF R**	HC	CP	0.58
		MCI	0.16
		CI	**< 0.001**
	CP	MCI	1
		CI	**< 0.001**
	MCI	CI	**< 0.001**
**ILF L**	HC	CP	0.91
		MCI	**0.040**
		CI	**< 0.001**
	CP	MCI	0.61
		CI	**< 0.001**
	MCI	CI	**0.002**
**ILF R**	HC	CP	1
		MCI	0.73
		CI	**< 0.001**
	CP	MCI	1
		CI	**< 0.001**
	MCI	CI	**< 0.001**

Supplementary Table 8. Post-hoc comparisons between cognitive profiles for FC at follow-up (next)

Tract of interest			P-value
**SLF L**	HC	CP	0.098
		MCI	0.44
		CI	**< 0.001**
	CP	MCI	1
		CI	**< 0.001**
	MCI	CI	**0.001**
**SLF R**	HC	CP	0.17
		MCI	1
		CI	**< 0.001**
	CP	MCI	1
		CI	**0.005**
	MCI	CI	**0.004**
**Uncinate fasciculus L**	HC	CP	1
		MCI	1
		CI	**0.003**
	CP	MCI	0.47
		CI	**< 0.001**
	MCI	CI	0.080
**Uncinate fasciculus R**	HC	CP	1
		MCI	1
		CI	**< 0.001**
	CP	MCI	1
		CI	**< 0.001**
	MCI	CI	**< 0.001**
**SLF (temporal part) R**	HC	CP	0.66
		MCI	0.20
		CI	**< 0.001**
	CP	MCI	1
		CI	**< 0.001**
	MCI	CI	**0.005**

FC: Fiber cross-section; HC: healthy controls; CP: cognitively preserved; MCI: mildly cognitively impaired; CI: cognitively impaired; L: left; R: right; IFOF: inferior fronto-occipital fasciculus; ILF: inferior longitudinal fasciculus; SLF: superior longitudinal fasciculus. All p-values are Bonferroni corrected and a p-value < 0.05 is considered significant.

Supplementary Table 9. Post-hoc comparisons between cognitive profiles for FDC at follow-up

**Tract of interest**			**P-value**
**Anterior thalamic radiation L**	HC	CP	**0.029**
		MCI	**0.009**
		CI	< **0.001**
	CP	MCI	1
		CI	< **0.001**
	MCI	CI	< **0.001**
**Anterior thalamic radiation R**	HC	CP	**0.002**
		MCI	< **0.001**
		CI	< **0.001**
	CP	MCI	1
		CI	< **0.001**
	MCI	CI	< **0.001**
**Corticospinal tract L**	HC	CP	**0.005**
		MCI	**0.001**
		CI	< **0.001**
	CP	MCI	1
		CI	< **0.001**
	MCI	CI	**0.001**
**Corticospinal tract R**	HC	CP	**0.020**
		MCI	**0.003**
		CI	< **0.001**
	CP	MCI	1
		CI	< **0.001**
	MCI	CI	< **0.001**
**Cingulum (cingulate gyrus) L**	HC	CP	**0.001**
		MCI	**0.002**
		CI	< **0.001**
	CP	MCI	1
		CI	< **0.001**
	MCI	CI	< **0.001**
**Cingulum (cingulate gyrus) R**	HC	CP	< **0.001**
		MCI	< **0.001**
		CI	< **0.001**
	CP	MCI	1
		CI	< **0.001**
	MCI	CI	< **0.001**
**Cingulum (hippocampus) L**	HC	CP	0.43
		MCI	**0.008**
		CI	< **0.001**
	CP	MCI	0.40
		CI	< **0.001**
	MCI	CI	**0.001**
**Cingulum (hippocampus) R**	HC	CP	**0.006**
		MCI	**0.001**
		CI	< **0.001**
	CP	MCI	1
		CI	< **0.001**
	MCI	CI	< **0.001**

Supplementary Table 9. Post-hoc comparisons between cognitive profiles for FDC at follow-up (next)

**Tract of interest**			**P-value**
**Forceps major**	HC	CP	**0.026**
		MCI	**0.001**
		CI	**< 0.001**
	CP	MCI	0.99
		CI	**< 0.001**
	MCI	CI	**< 0.001**
**Forceps minor**	HC	CP	0.10
		MCI	1
		CI	**< 0.001**
	CP	MCI	1
		CI	**< 0.001**
	MCI	CI	**0.002**
**IFOF L**	HC	CP	0.37
		MCI	0.12
		CI	**< 0.001**
	CP	MCI	1
		CI	**< 0.001**
	MCI	CI	**< 0.001**
**IFOF R**	HC	CP	0.079
		MCI	**0.021**
		CI	**< 0.001**
	CP	MCI	1
		CI	**< 0.001**
	MCI	CI	**< 0.001**
**ILF L**	HC	CP	0.051
		MCI	**0.004**
		CI	**< 0.001**
	CP	MCI	1
		CI	**< 0.001**
	MCI	CI	**< 0.001**
**ILF R**	HC	CP	0.069
		MCI	**0.023**
		CI	**< 0.001**
	CP	MCI	1
		CI	**< 0.001**
	MCI	CI	**< 0.001**
**SLF L**	HC	CP	**0.012**
		MCI	**0.037**
		CI	**< 0.001**
	CP	MCI	1
		CI	**< 0.001**
	MCI	CI	**< 0.001**
**SLF R**	HC	CP	**0.005**
		MCI	**0.029**
		CI	**< 0.001**
	CP	MCI	1
		CI	**< 0.001**
	MCI	CI	**0.001**

Supplementary Table 9. Post-hoc comparisons between cognitive profiles for FDC at follow-up (next)

**Tract of interest**			**P-value**
**Uncinate fasciculus L**	HC	CP	0.44
		MCI	0.051
		CI	**< 0.001**
	CP	MCI	1
		CI	**< 0.001**
	MCI	CI	**0.003**
**Uncinate fasciculus R**	HC	CP	0.16
		MCI	**0.018**
		CI	**< 0.001**
	CP	MCI	1
		CI	**< 0.001**
	MCI	CI	**< 0.001**
**SLF (temporal part) L**	HC	CP	0.076
		MCI	0.11
		CI	**< 0.001**
	CP	MCI	1
		CI	**< 0.001**
	MCI	CI	**0.003**
**SLF (temporal part) R**	HC	CP	**0.021**
		MCI	**0.022**
		CI	**< 0.001**
	CP	MCI	1
		CI	**< 0.001**
	MCI	CI	**0.007**

FDC: Fiber density and cross-section; HC: healthy controls; CP: cognitively preserved; MCI: mildly cognitively impaired; CI: cognitively impaired; L: left; R: right; IFOF: inferior fronto-occipital fasciculus; ILF: inferior longitudinal fasciculus; SLF: superior longitudinal fasciculus. All p-values are Bonferroni corrected and a p-value < 0.05 is considered significant.

Supplementary Figure 1. Fiber density comparisons between clinical phenotypes for significant tracts at baseline

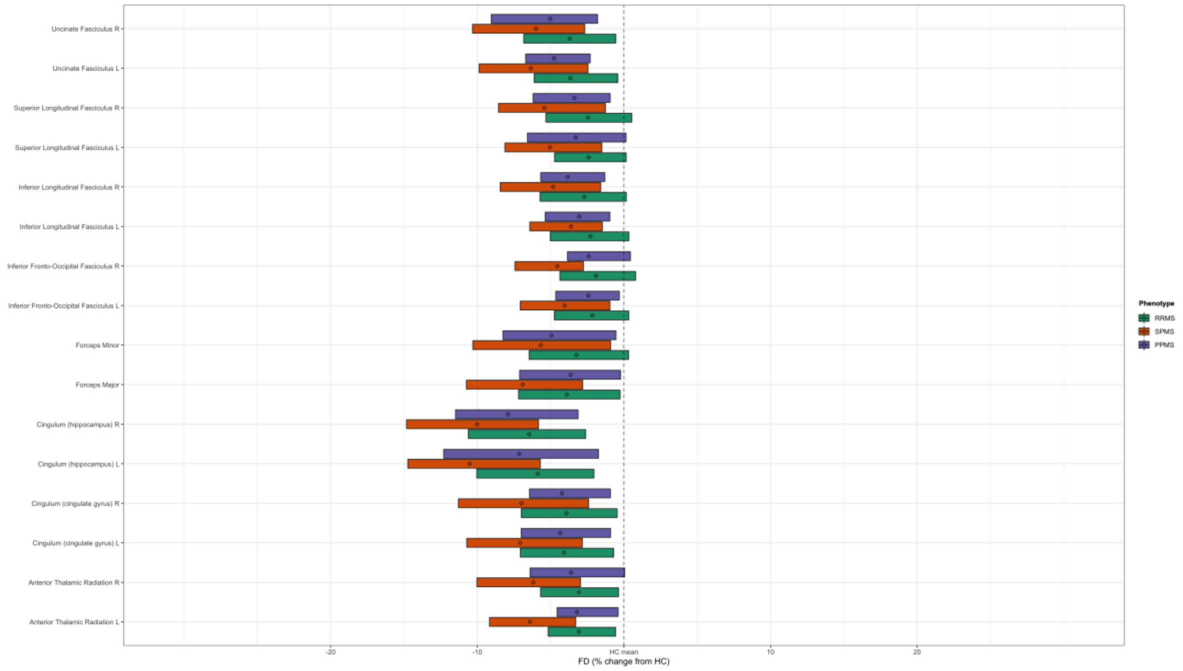

Supplementary Figure 2. Fiber cross-section comparisons between clinical phenotypes for significant tracts at baseline

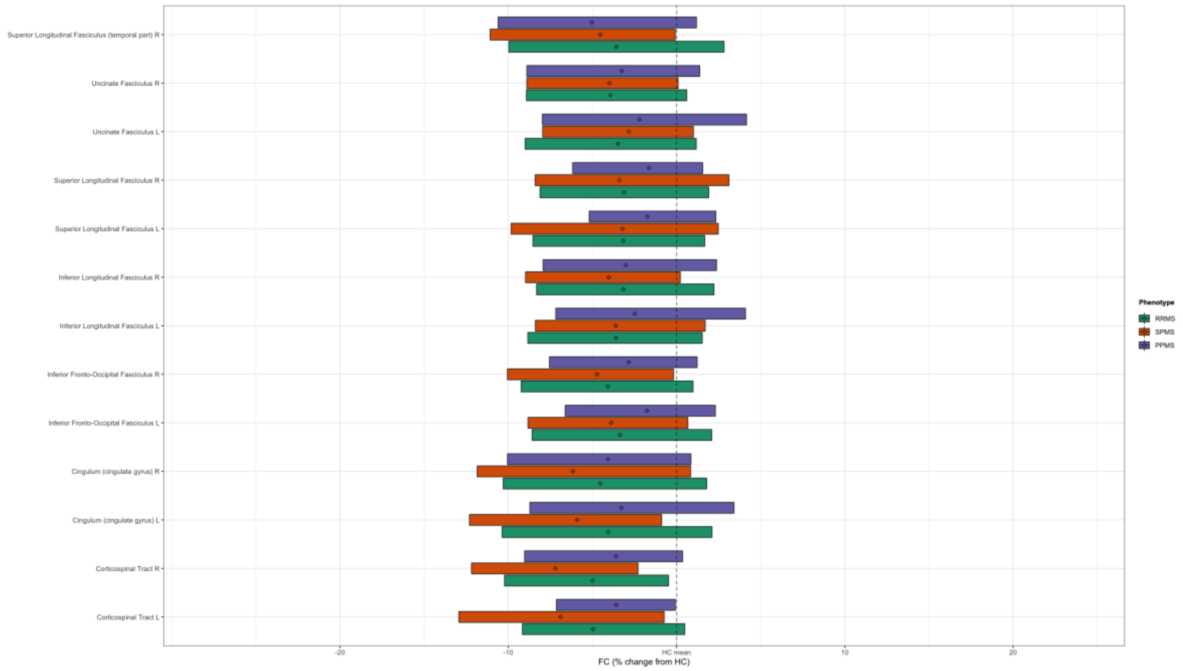

Supplementary Figure 3. Fiber density comparisons between clinical phenotypes for significant tracts at baseline (after discarding white matter lesions)

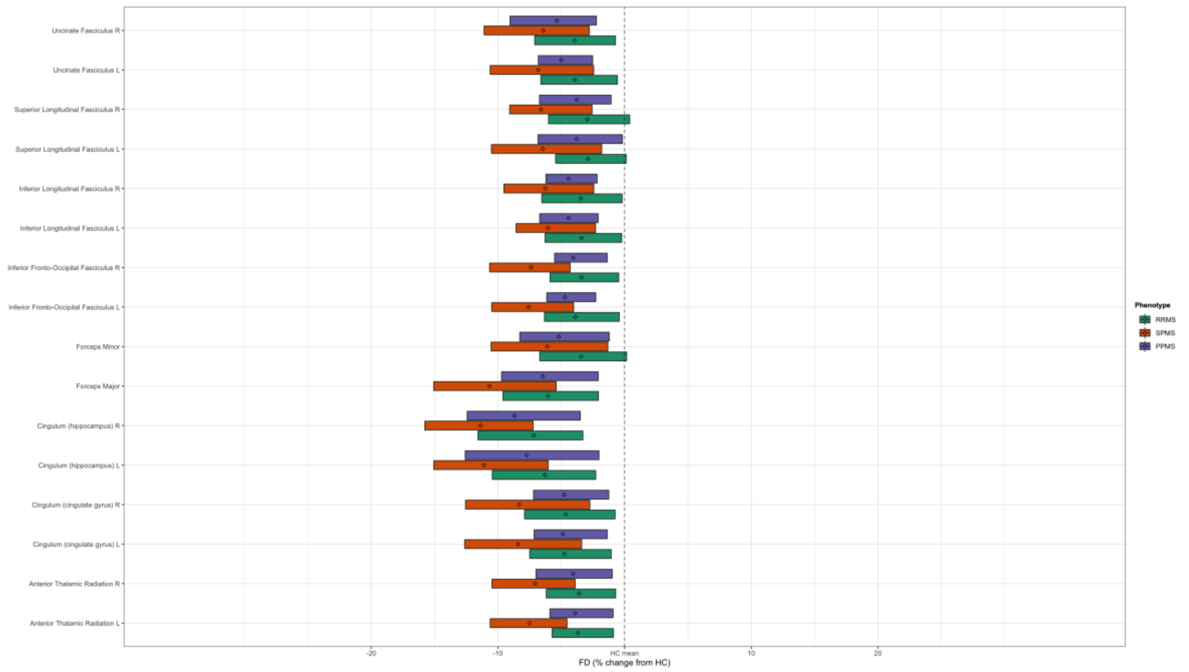

Supplementary Figure 4. Fiber cross-section comparisons between clinical phenotypes for significant tracts at baseline (after discarding white matter lesions)

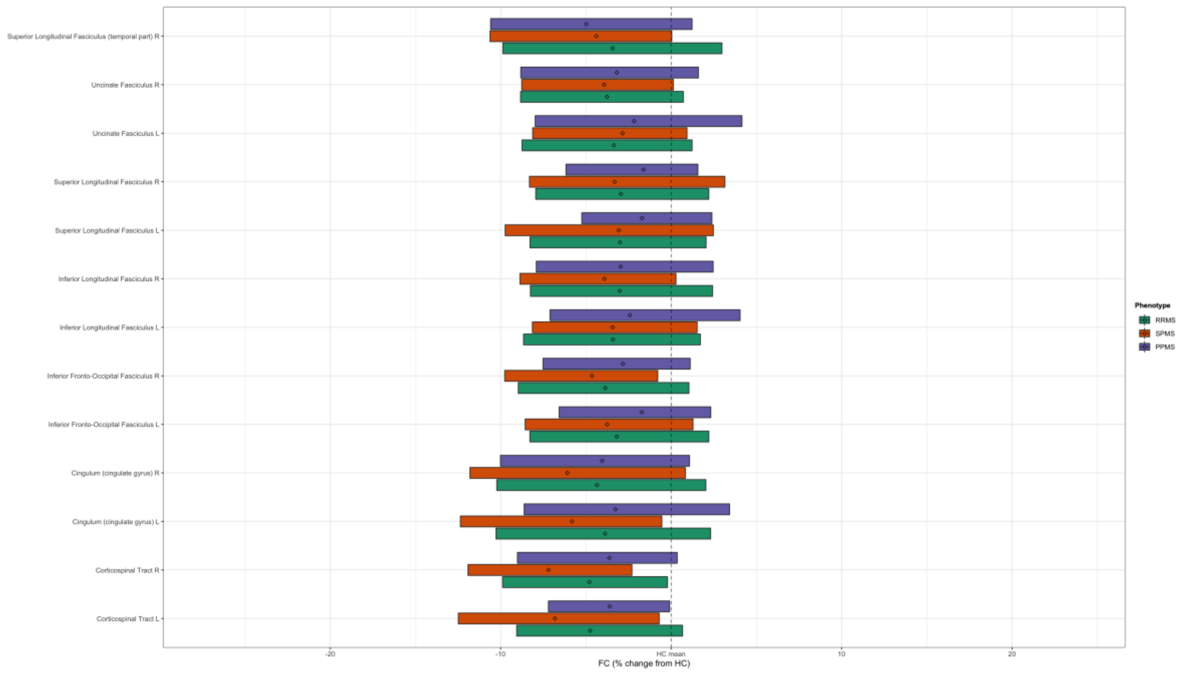

Supplementary Figure 5. Fiber density and cross-section comparisons between clinical phenotypes for significant tracts at baseline (after discarding white matter lesions)

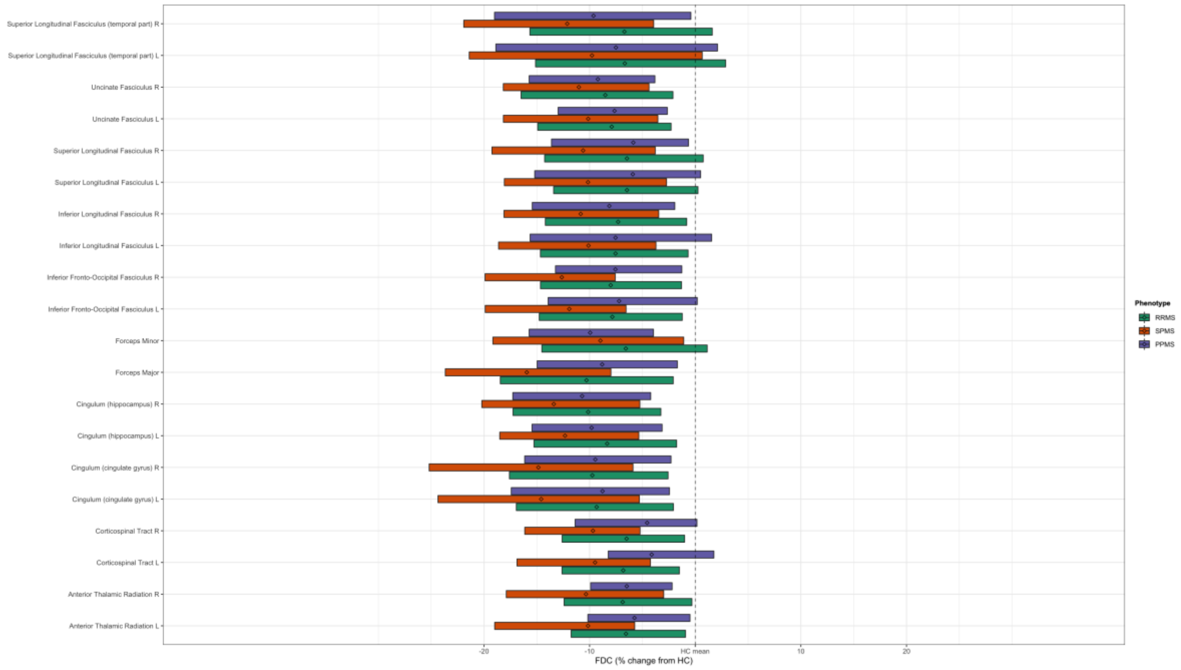

Supplementary Figure 6. Longitudinal changes of fiber density by clinical phenotype for significant tracts

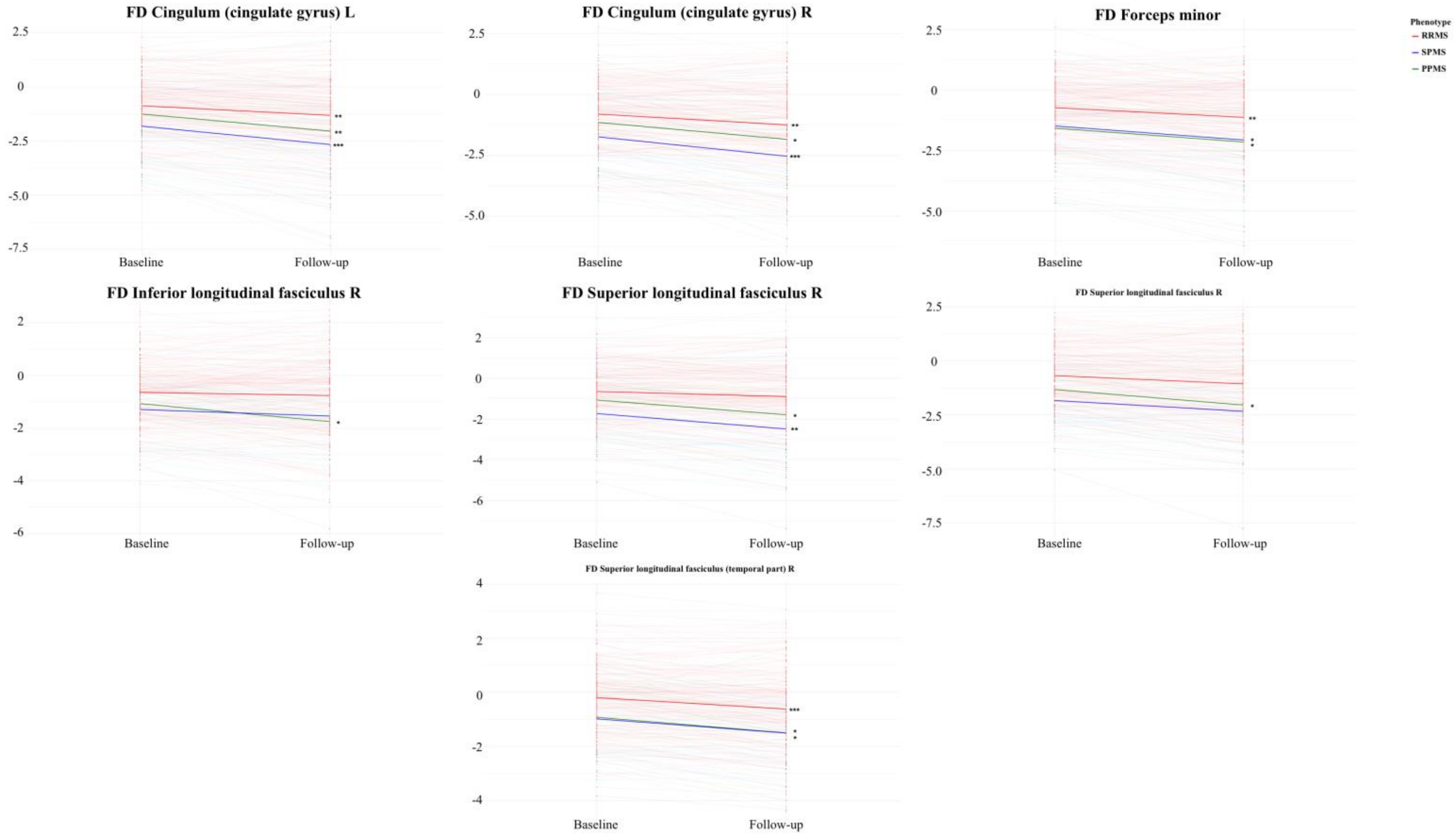

Supplementary Figure 7. Longitudinal changes of fiber cross-section by clinical phenotype for significant tracts

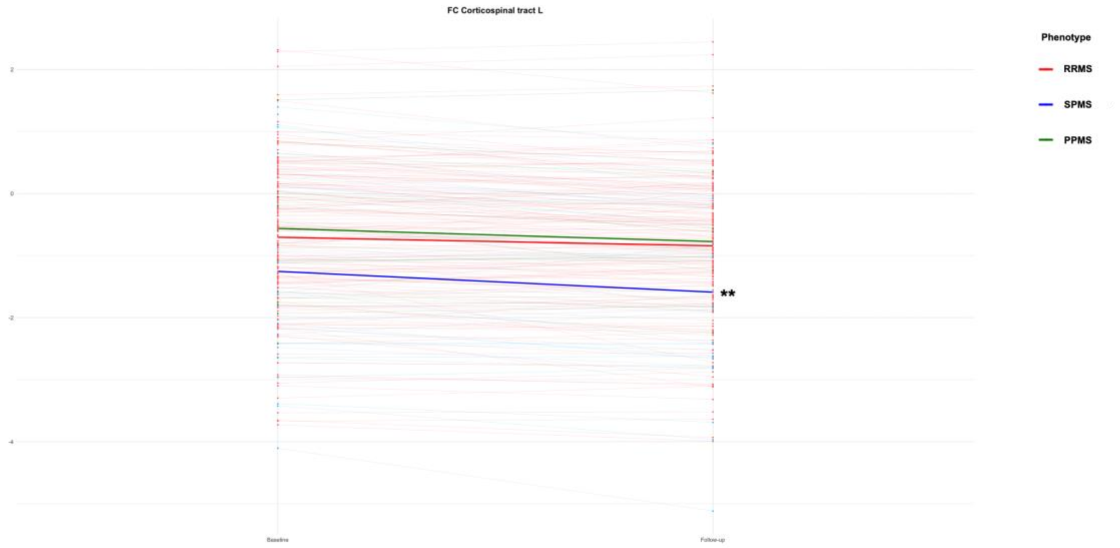

Supplementary Figure 8. Longitudinal changes of fiber density and cross-section by clinical phenotype for significant tracts

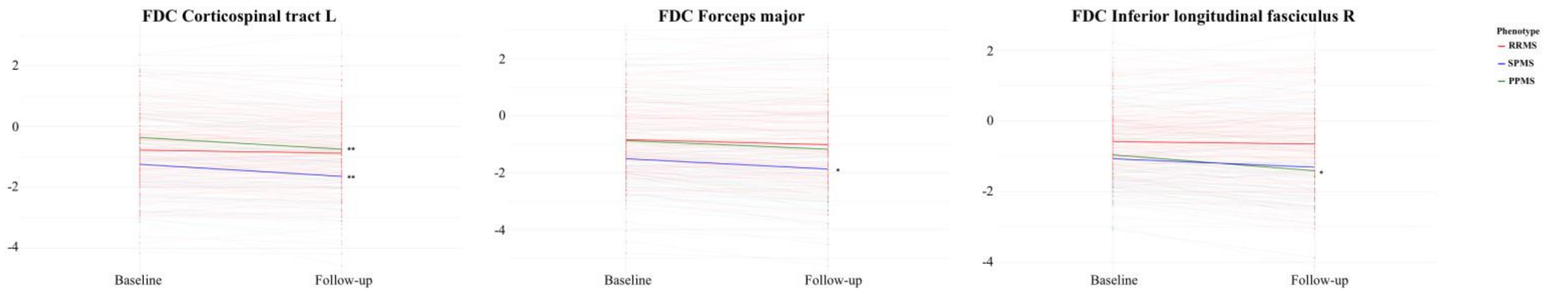

Supplementary Figure 9. Longitudinal changes of fiber density by clinical phenotype for significant tracts (after discarding white matter lesions)

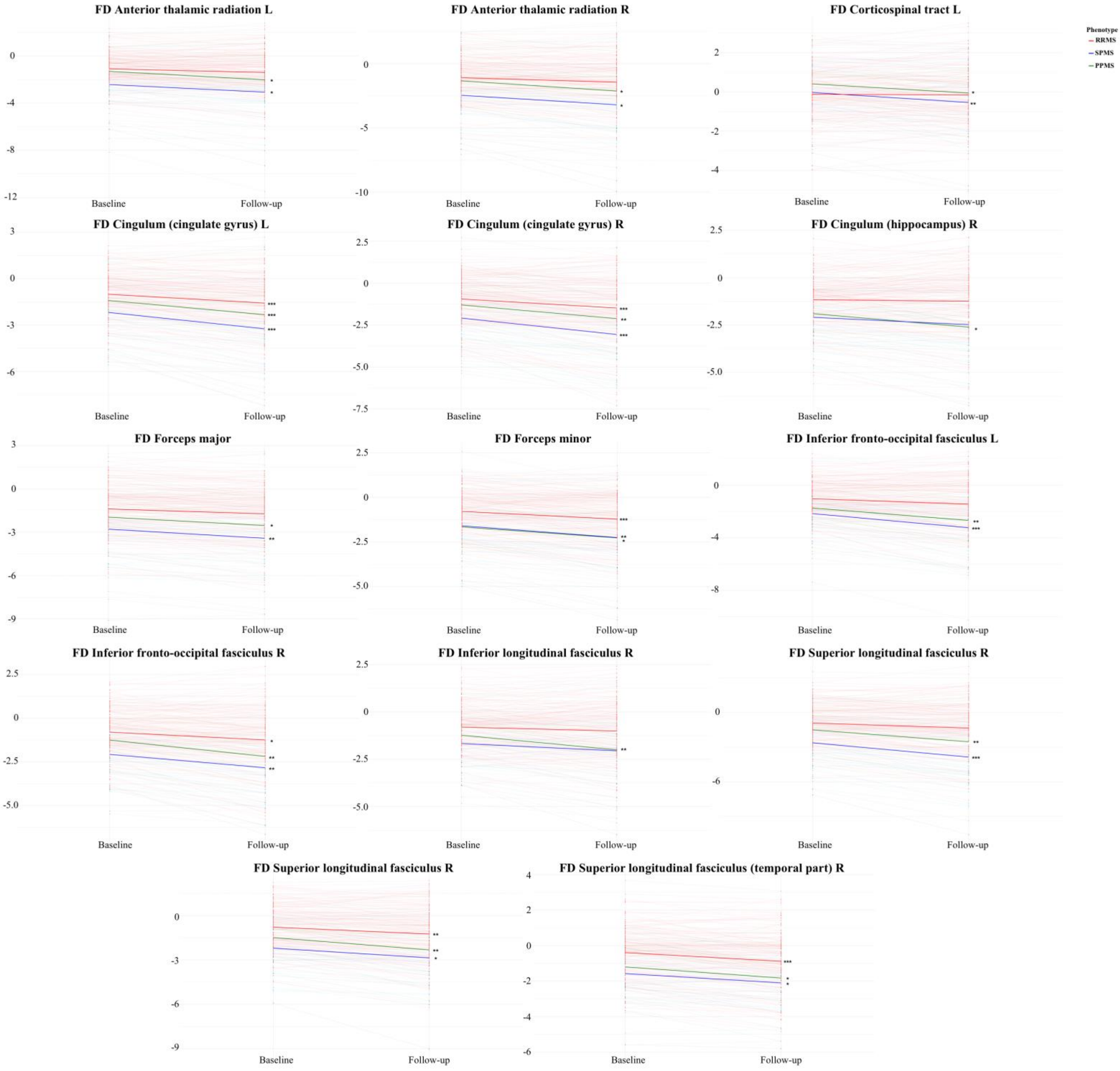

Supplementary Figure 10. Longitudinal changes of fiber cross-section by clinical phenotype for significant tracts (after discarding white matter lesions)

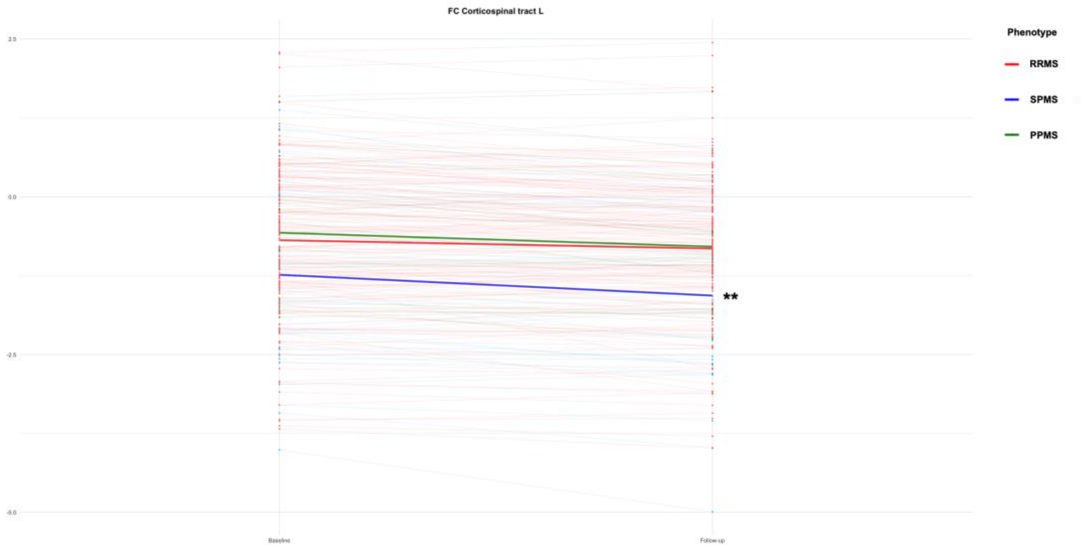

Supplementary Figure 11. Fiber density comparisons between cognitive profiles for significant tracts at baseline

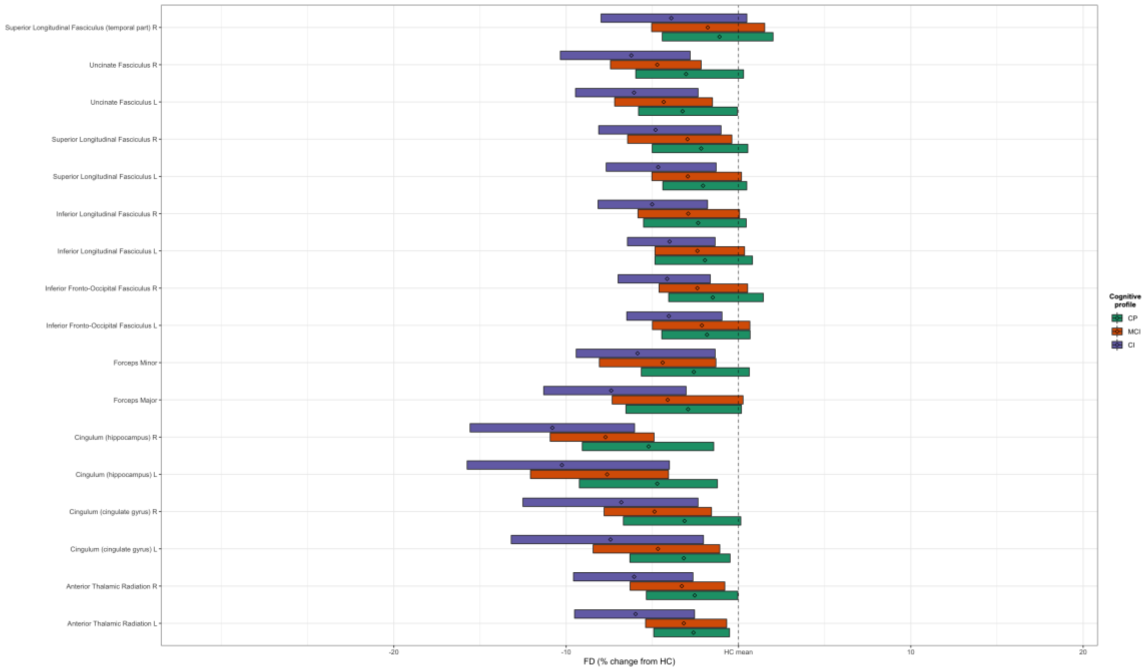

Supplementary Figure 12. Fiber cross-section comparisons between cognitive profiles for significant tracts at baseline

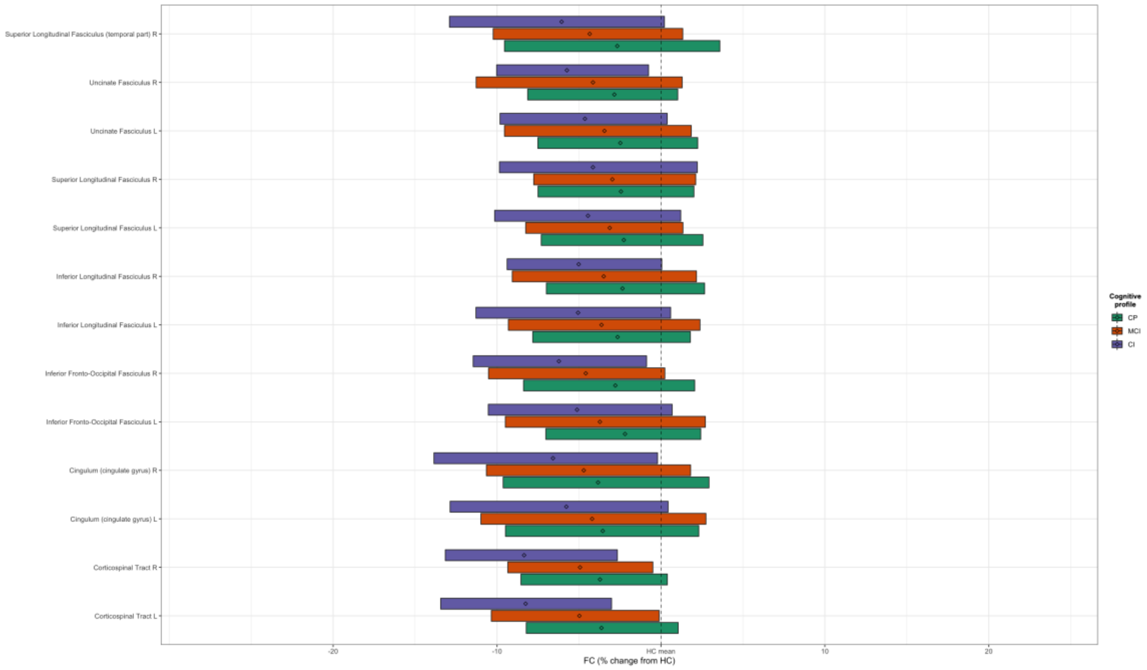

Supplementary Figure 13. Fiber density comparisons between cognitive profiles for significant tracts at baseline (after discarding white matter lesions)

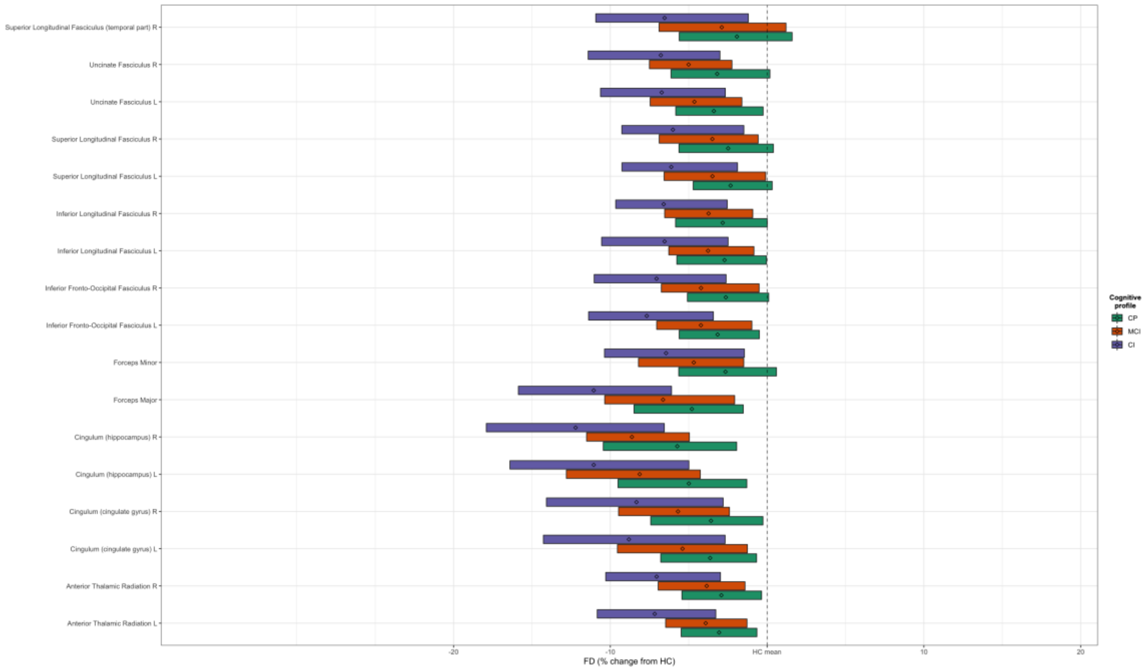

Supplementary Figure 14. Fiber cross-section comparisons between cognitive profiles for significant tracts at baseline (after discarding white matter lesions)

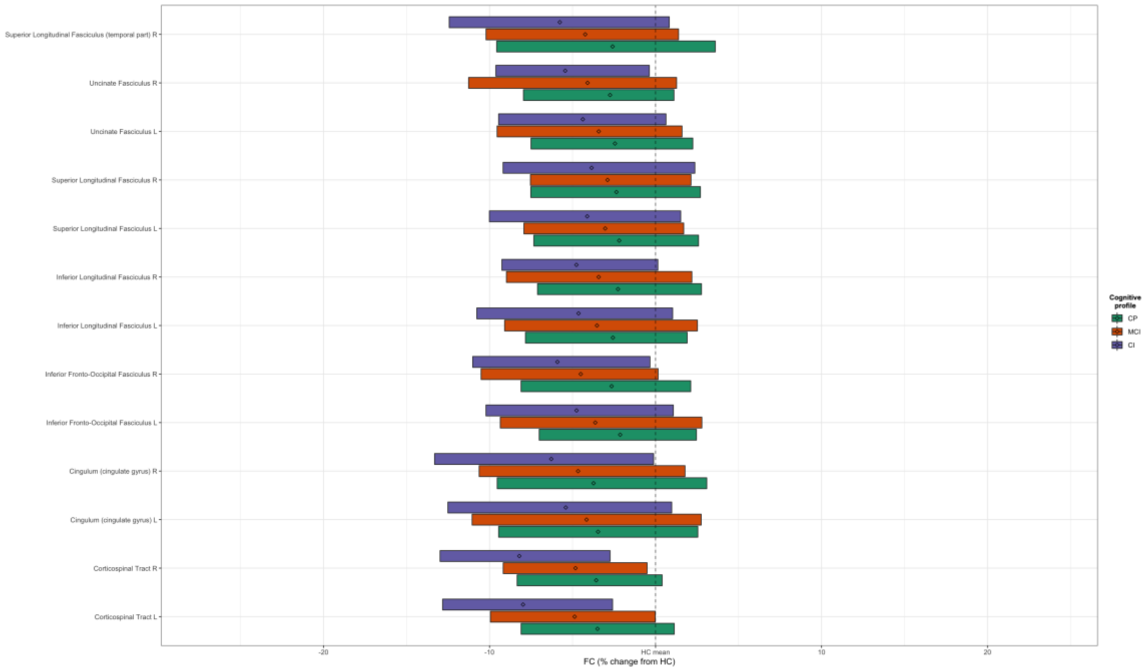

Supplementary Figure 15. Fiber density and cross-section comparisons between cognitive profiles for significant tracts at baseline (after discarding white matter lesions)

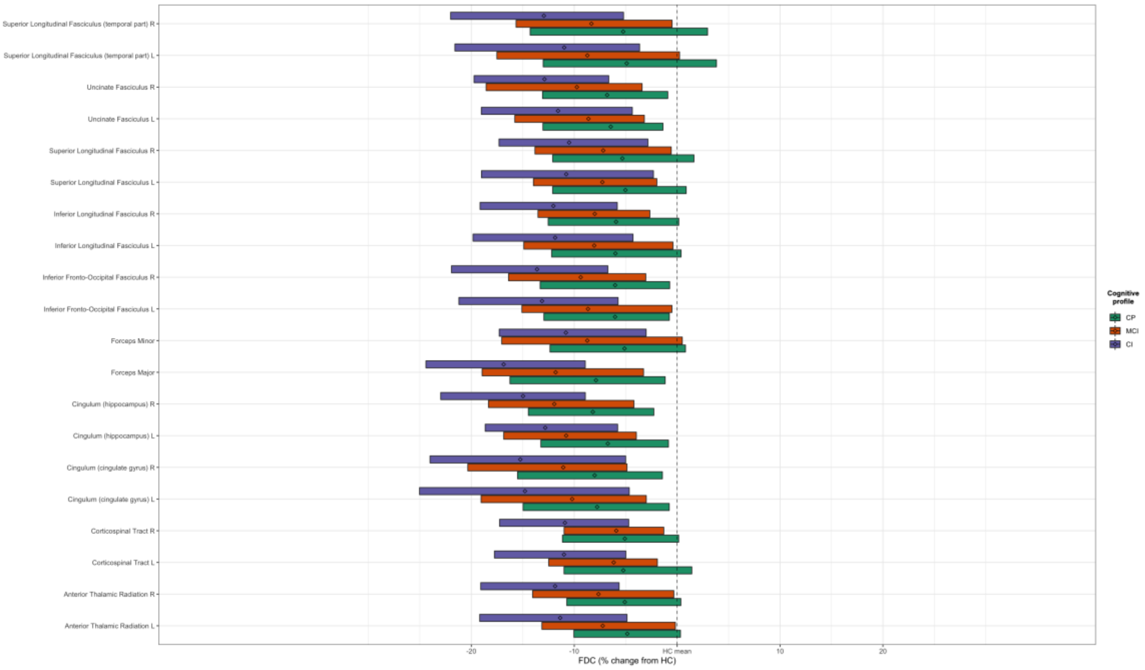

Supplementary Figure 16. Fiber density comparisons between cognitive profiles for significant tracts at follow-up

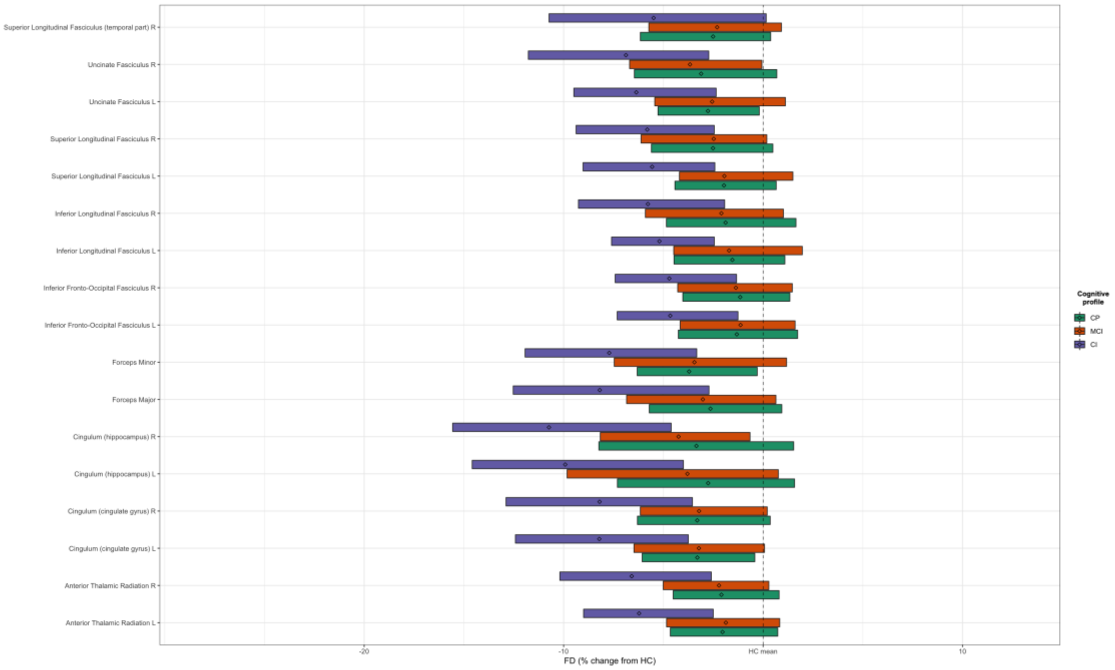

Supplementary Figure 17. Fiber cross-section comparisons between cognitive profiles for significant tracts at follow-up

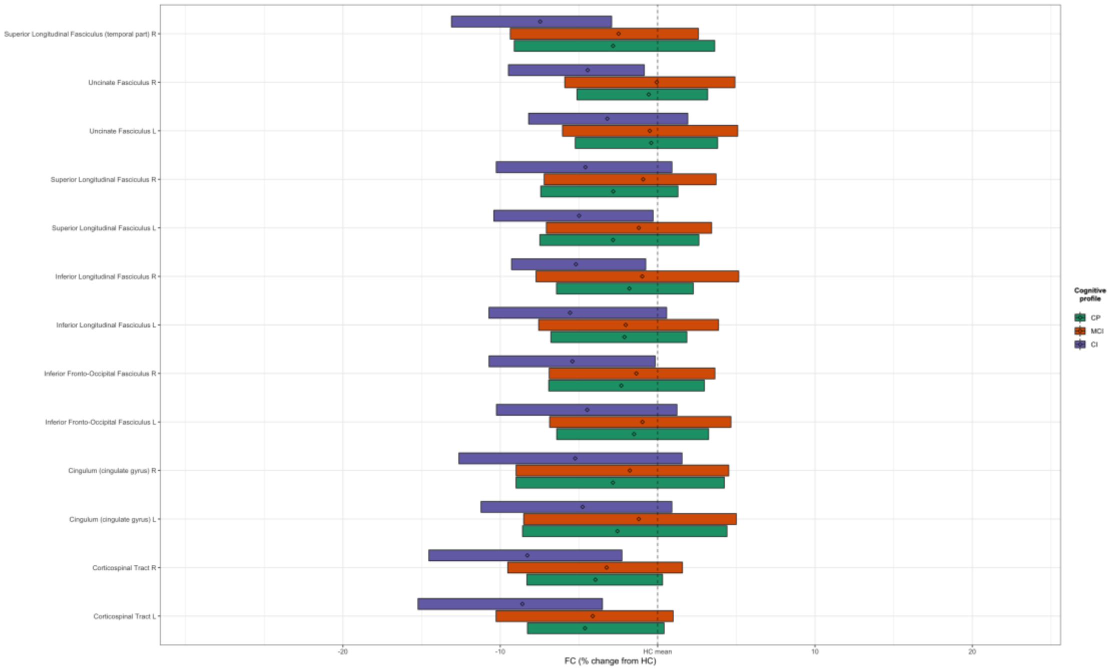

Supplementary Figure 18. Fiber density comparisons between cognitive profiles for significant tracts at follow-up (after discarding white matter lesions)

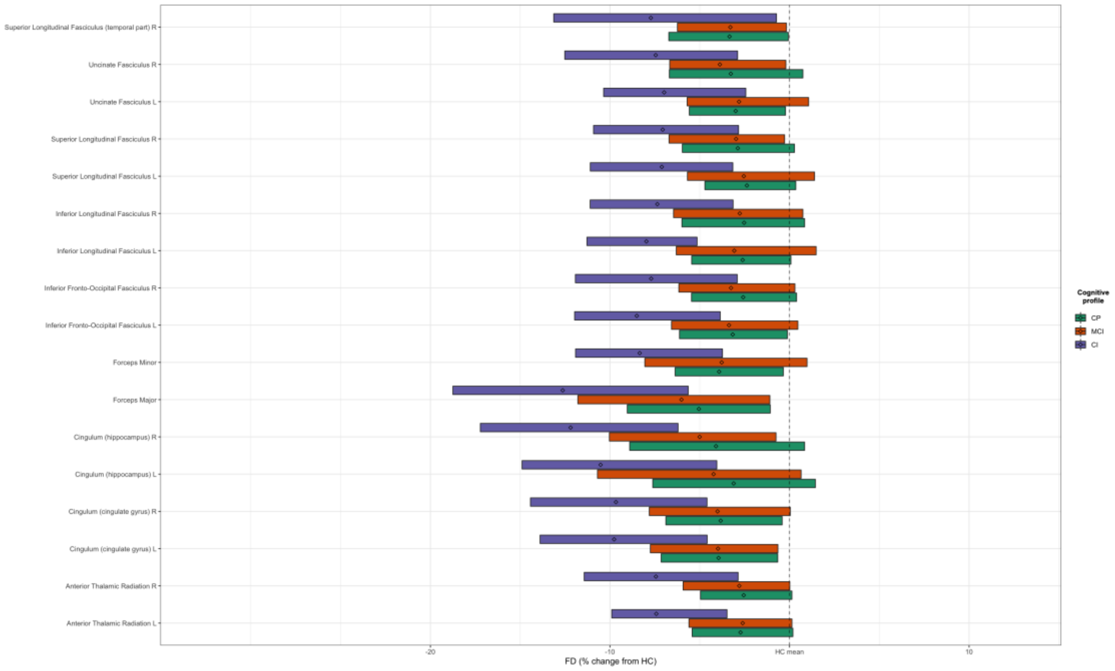

Supplementary Figure 19. Fiber cross-section comparisons between cognitive profiles for significant tracts at follow-up (after discarding white matter lesions)

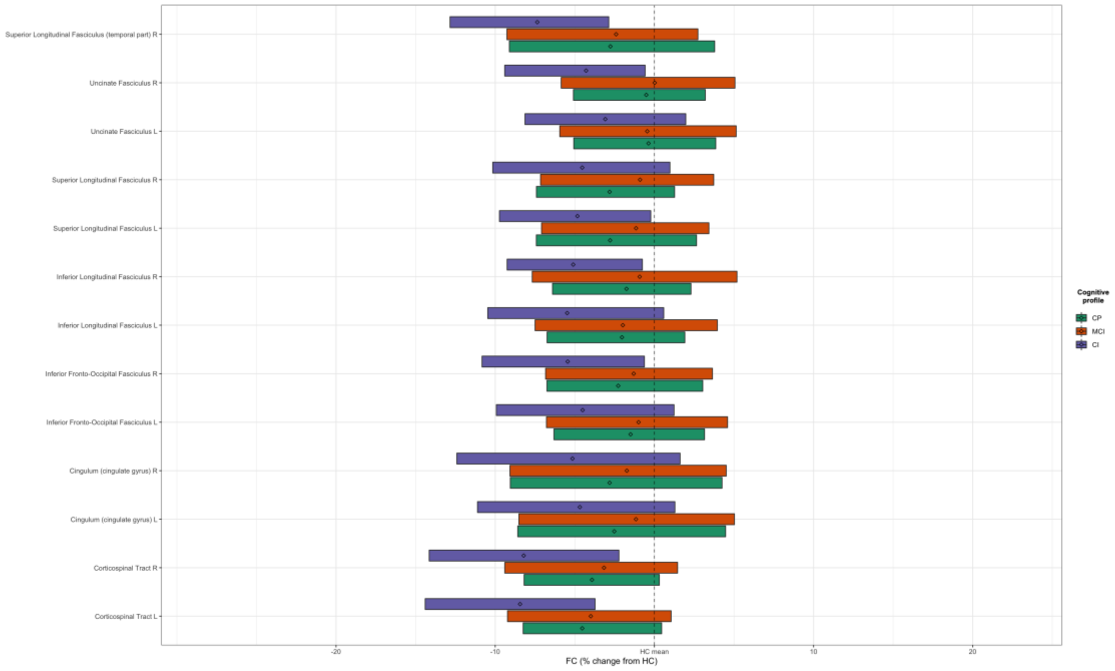

Supplementary Figure 20. Fiber density and cross-section comparisons between cognitive profiles for significant tracts at follow-up (after discarding white matter lesions)

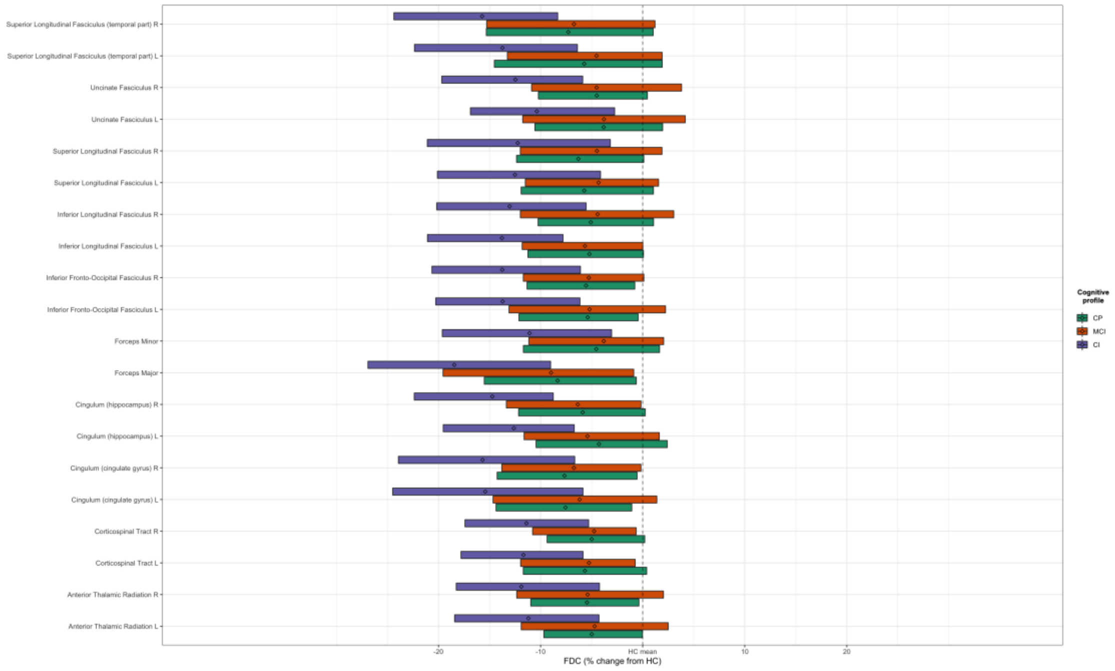

Supplement: fcae018_Supplementary_Data [file fcae018_supplementary_data.pdf]
